# Microbial Surface Glycan Probe Isolates Anti‑l‑Rhamnose Antibodies from Human Serum for Bacterial Detection

**DOI:** 10.1021/acsinfecdis.5c00757

**Published:** 2025-11-25

**Authors:** Hersa Milawati, Mia Sheshova, Joanna Joo, Tania J. Lupoli

**Affiliations:** Department of Chemistry, 5894 New York University, New York, New York 10003, United States

**Keywords:** Rare sugars, bacteria, rhamnose, human serum, anti-glycan antibodies

## Abstract

Bacterial strains are distinguished by surface glycans composed of defined sugar sequences that include “rare” monosaccharides, which are absent in human glycans and help to mediate host–microbe interactions. One of the most prevalent rare sugars is l-Rhamnose (l-Rha), and human sera are generally enriched in anti-l-Rha antibodies; however, the source of l-Rha antigens is unknown. Here, we synthesize a surface glycan l-Rha-*N*-acetyl glucosamine disaccharide sequence, which is found across many bacterial species, to evaluate binding motifs of human anti-glycan antibodies in clinical and commercial human sera. We find that sera are enriched in IgG antibodies that react with this disaccharide probe. Through capture of bound antibodies and analysis with surface glycan sequences from different strains, we observe that bound human antibodies appear to recognize free or branched, but not internal, l-Rha motifs. Overall, this work details the isolation of naturally occurring anti-l-Rha human antibodies and promotes an understanding of their carbohydrate recognition epitopes.

## Graphical abstract

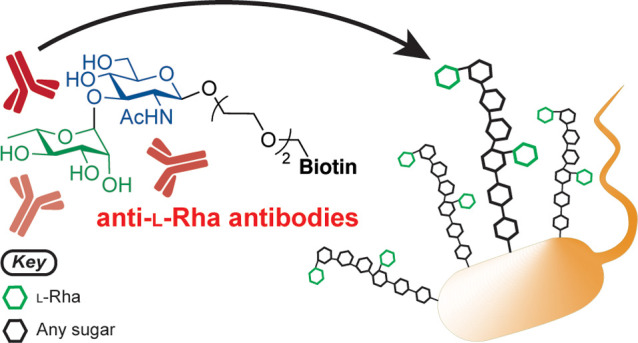

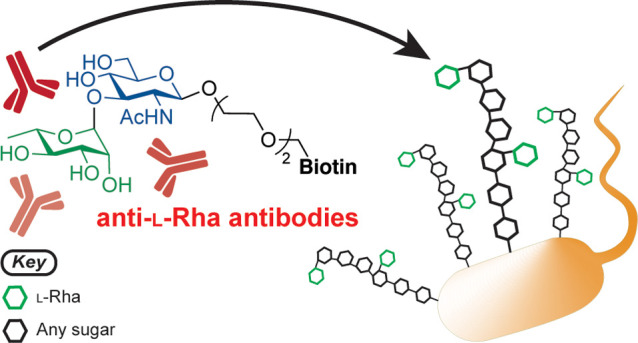

Bacterial cell surfaces are decorated with distinct sequences of sugars that facilitate molecular interactions with the environment and help to distinguish different strains (Figure 1A). Across bacteria, hundreds of monosaccharide building blocks are used for the biosynthesis of different glycans, while approximately ten distinct monosaccharides are used by humans.^1–3^ Those sugars that are absent in mammals, but present in microbes and plants, are known as “rare”.^1,4^ One of the most common rare monosaccharides is a C6-deoxysugar called l-rhamnose (l-Rha).^5,6^ l-Rha is present in a variety of plant and microbial glycoconjugates,^6^ including natural products, glycoproteins, polysaccharides, and other cell surface glycans. Given their absence in mammals, l-Rha and other rare sugars are believed to be antigenic. Accordingly, analyses of random human serum samples has revealed that, among panels of carbohydrate antigens, antibodies against α-l-Rha motifs on glycoconjugates are the most abundant.^7,8^ As a result, l-Rha has been used for endogenous anti-glycan antibody recruitment for a series of applications in eukaryotic cells.^4,9,10^ However, the mode of human antibody recognition of l-Rha within naturally occurring microbial glycans is not well-understood.

**Figure 1:**
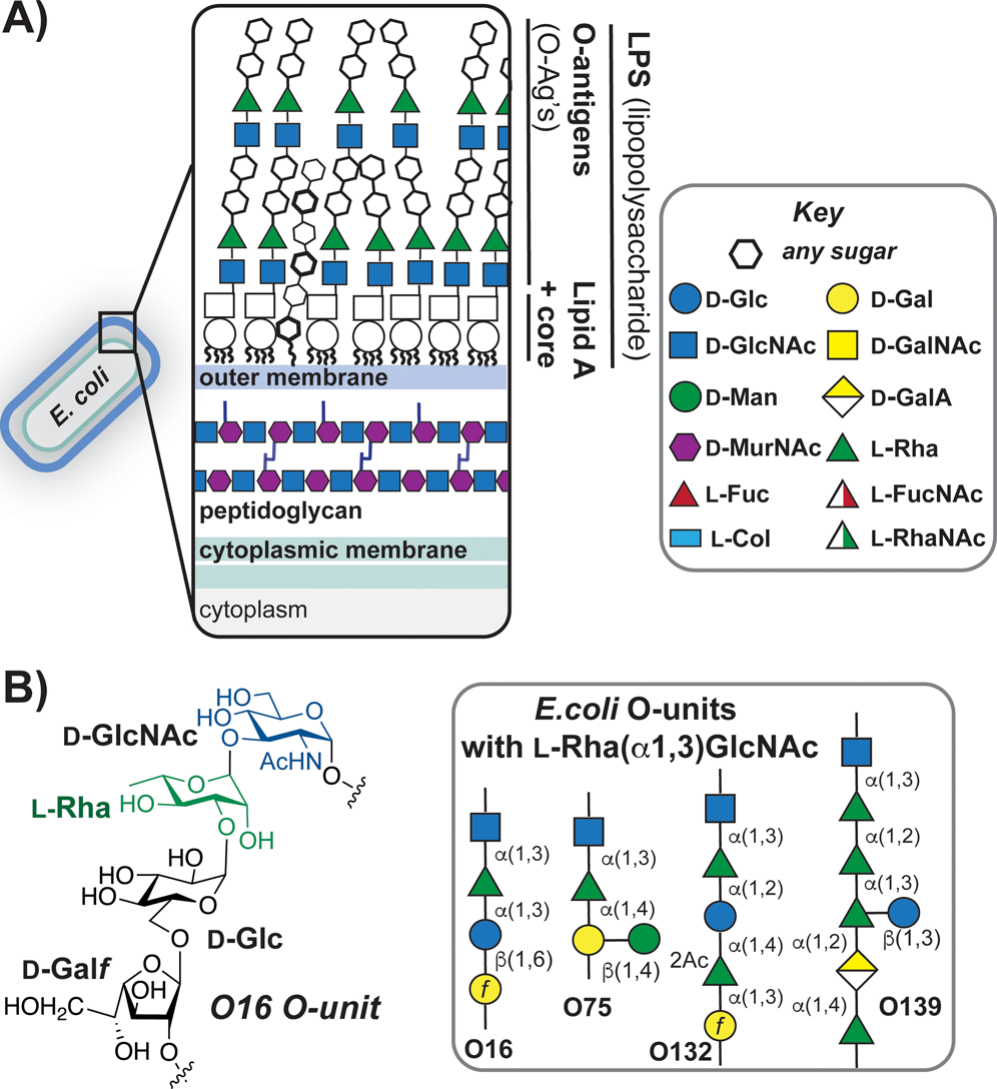
Bacterial cell surfaces are enriched in the rare sugar l-Rha. (A) Schematic of an *E. coli* cell envelope containing LPS with attached O-Ags composed of repeating O-units, which make-up the surface of the outer membrane. (B) Chemical structure of well-studied O16 O-Ag O-unit sequence and glycan symbols for select *E. coli* O-units that contain the same l-Rha­(α1,3)­GlcNAc disaccharide, which is found across other bacterial species as well. Note that some O16 sequences can contain a branched Glc residue and/or an acetylated l-Rha (l-Rha2Ac).^12^

In the bacterium *Escherichia coli*, almost 200 different strains can be discriminated based on the monosaccharide sequence of surface polysaccharides called O-antigens (O-Ags) that are attached to the distal portion of lipopolysaccharide (LPS) in the outer membrane of the cell envelope.^11,12^ These polysaccharides are important virulence factors that protect the cell from host defense tactics, namely, complement-mediated death in serum. O-Ags are built from repeating oligosaccharide units (O-units) containing 2–7 sugars.^2,11^ One of the best studied O-Ag biosynthetic pathways in *E. coli* is that of O16, which is composed of an O-unit with the following sugar sequence: galactofuranose­(β1,6)­glucose­(α1,3)l-Rha­(α1,3)­N-acetylglucosamine (Gal*f*(β1,6)­Glc­(α1,3)l-Rha­(α1,3)­GlcNAc)^12,13^ (Figure 1B). Several other O-Ag sequences in *E. coli* contain the disaccharide l-Rha­(α1,3)­GlcNAc, including O75, O132, O139, and O150.^12^ Notably, *E. coli* O75 strains are pathogenic and can cause bacteremia.^14^ Some *Salmonella* strains also express related O-Ags that contain the l-Rha­(α1,3)­GlcNAc disaccharide sequence, including SO11 and SO59,^15^ along with *Shigella* serotype F1-5.^16^ This same disaccharide is also found in mycobacterial cell envelopes and polysaccharides that extend from Gram-positive bacteria.^17,18^

Glycans found on the surfaces of bacteria, viruses, and parasites have garnered much attention as vaccine targets.^19^ Following the inception of synthetic vaccine conjugates, bacterial surface glycan fragments containing rare sugars have been demonstrated to represent key epitopes for recognition by antibodies.^19,20^ While isolated microbial polysaccharides can be used as antigens, glycans from natural sources are often complex and heterogeneous and can only be obtained from fermentable microbes.^19^ Hence, there has been much motivation to establish synthetic routes to minimal glycan fragments that represent immunogenic epitopes for the rational design of carbohydrate-based vaccines. Past work has shown that polysaccharide fragments,^20,21^ including disaccharide probes,^22^ are sufficient for activation of an immune response. Taken together, these observations led to our postulation that the l-Rha­(α1,3)­GlcNAc disaccharide may serve as a distinct motif for the detection of various bacterial strains by host proteins.

Here, we developed a synthetic route to a biotinylated probe containing the bacterial disaccharide l-Rha­(α-1,3)­GlcNAc. We hypothesized that, because human serum is known to be enriched in anti-l-Rha antibodies and this disaccharide is found in and on many naturally occurring bacteria, we could isolate antibodies that react with our probe from existing sera and study the interactions of these antibodies with microbial glycans. To do so, we first used an enzyme-linked immunosorbent assay (ELISA) format to assess detection of this probe by both commercial and clinical human serum samples. Pull-down protocols were then optimized to isolate human antibodies bound to the disaccharide probe, and the resulting antibody mixtures were then used to evaluate the detection of various microbial strains, leading to new observations about the types of l-Rha motifs recognized by human sera.

We envisioned the target synthetic l-Rha­(α1,3)­GlcNAc biotinylated probe (“**RGB**”, **1**) to be derived from four fragments, rhamnosyl donor **2**, glucosamine acceptor **3**, and azido polyethylene glycol (PEG) linker **4** for a series of stepwise glycosylation reactions, along with biotin *N*-hydroxysuccinimide (NHS) ester (Scheme 1).

**Scheme 1:**
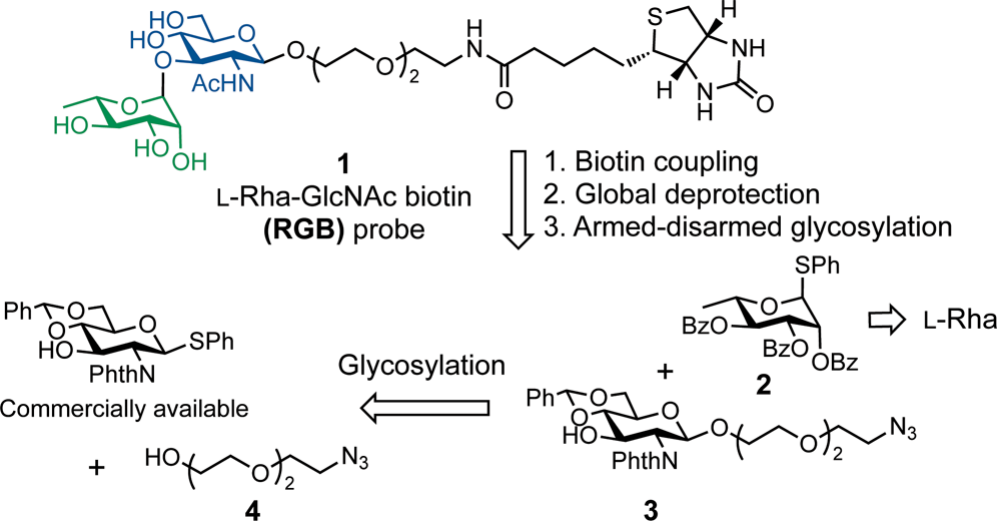
Retrosynthetic Scheme for Rare Sugar-Containing Probe **1** (**RGB**)­^a^

a Ac = acetyl, Ph = phenyl, Bz = benzoyl, and Phth = phthaloyl.

To synthesize probe **1**, l-Rha was first converted into thiorhamnoside **2** in four steps by peracetylation, thioglycosylation, Zemplén deacetylation,^23^ and perbenzoylation (Scheme S2A). Briefly, peracetylated l-Rha was treated with thiophenol and boron trifluoride etherate to give **5** as predominantly the α-anomer. Zemplén’s method of deacetylation followed using sodium methoxide in methanol. Subsequent benzoylation afforded us the desired perbenzoylated thio-α-rhamnoside **2** in good yield. Isolation of the α-isomer was indicated by a ^3^ *J*_1,2_ coupling constant of 1.6 Hz (Table S1). It should be noted that Ghosh and co-workers reported attempted glycosylation reactions with the peracetylated rhamnoside **5**, but these reactions failed to give the expected product under several different conditions.^24^ For this reason, we performed the described protecting group manipulation to obtain rhamnoside **2**.

The first glycosylation was carried out between a commercially available glucosamine donor and an azido-PEG_3_-alcohol linker (**4**, Scheme 2B), the latter of which was synthesized based on a reported method by Wang and co-workers.^25^ Glycosylation in the presence of *N*-iodosuccinimide (NIS) and trimethylsilyl triflate (TMSOTf) as an activator provided the corresponding glycosylated product **3** in a β-selective manner, as confirmed by a ^3^ *J*_1,2_ coupling constant of 8.4 Hz (Table S1).

**Scheme 2:**
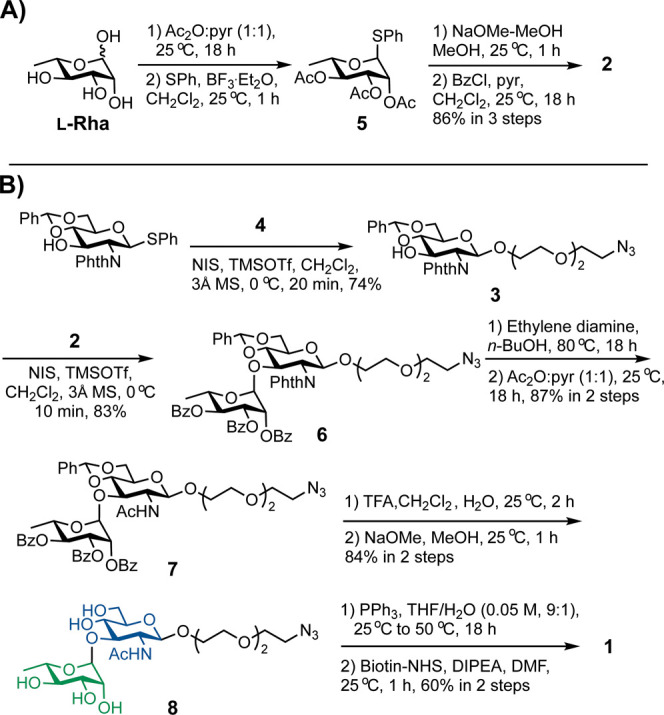
Synthetic Scheme to Obtain Probe **1** ^a^

a Ac_2_O = acetic anhydride, pyr = pyridine, Ph = phenyl, BF_3_·Et_2_O = boron trifluoride etherate, NaOMe = sodium methoxide, MeOH = methanol, BzCl = benzoyl chloride, Phth = phthaloyl, NIS = *N*-iodosuccinimide, TMSOTf = trimethylsilyl trifluoromethanesulfonate, MS = molecular sieves, *n*-BuOH = *n*-butanol, TFA = trifluoroacetic acid, PPh_3_ = triphenylphosphine, THF = tetrahydrofuran, Biotin-NHS = biotin *N*-hydroxysuccinimide ester, and DIPEA = diisopropylethylamine, DMF = dimethylformamide.

The second glycosylation to produce protected disaccharide **6** was then carried out with **3** as the acceptor and thiorhamnoside **2** as the donor. The glycosylation reaction progressed rapidly using the NIS/TMSOTf activation system after several rounds of condition optimization. Namely, the yield was improved by increasing the amount of donor (see Table S2). Compound **6** had two observable anomeric protons, one at δ 5.25 ppm (d, ^3^ *J*_1,2_ = 8.4 Hz) and one at δ 4.74 ppm (d, ^3^ *J*_1,2_ = 1.6 Hz) (Table S1), the latter of which indicated the formation of a new α-linkage between the protected sugars.

Finally, we performed serial global deprotection starting with removal of *N*-phthalimide under basic conditions at an elevated temperature, followed by acetylation to obtain compound **7** in a high yield. Then, hydrolysis under acidic conditions and a Zemplén deprotection^23^ removed the benzylidene and benzoyl groups, respectively, to afford compound **8**. Reduction of the azide group was carried out using triphenylphosphine, and the crude product was immediately coupled to a biotin-NHS ester under basic conditions to ultimately provide milligram quantities of the desired product **1** (Table S1). It should be noted that the assignment of anomeric positions in compounds **6**, **7**, **8**, and **1** was supported by heteronuclear single quantum coherence (HSQC) analysis.

With our **RGB** probe in hand, we moved on to assess if it could be employed to detect antibodies in different human serum samples using streptavidin-coated plates (Figure 2A). As IgA, IgG, and IgM are the most common isotypes in human serum,^26^ we analyzed the relative abundance of each that bound to the probe. We observed that both commercial and clinical human serum samples were enriched in anti-**RGB** IgG antibodies compared to the control wells containing only biotin (Figure 2B). While we detected **RGB**-reactive IgA and IgM across commercial human serum samples, including samples purified for single antibody isotypes, their levels were not as abundant as IgG across all clinical samples (Figures 2B and S1). It should be noted that human IgG antibody reactivity with synthetic l-Rha has been observed across many other human serum samples.^9^ Further, IgG is the most abundant isotype in serum;^26^ higher affinity anti-glycan IgG can be produced as a result of antigen exposure,^9,26,27^ and human IgG antibody reactivity to microbial glycans is a well-documented benchmark for vaccine candidate evaluation, as IgGs can promote complement-mediated toxicity.^20–22,28^ Hence, we continued to analyze IgG levels in further experiments.

**Figure 2:**
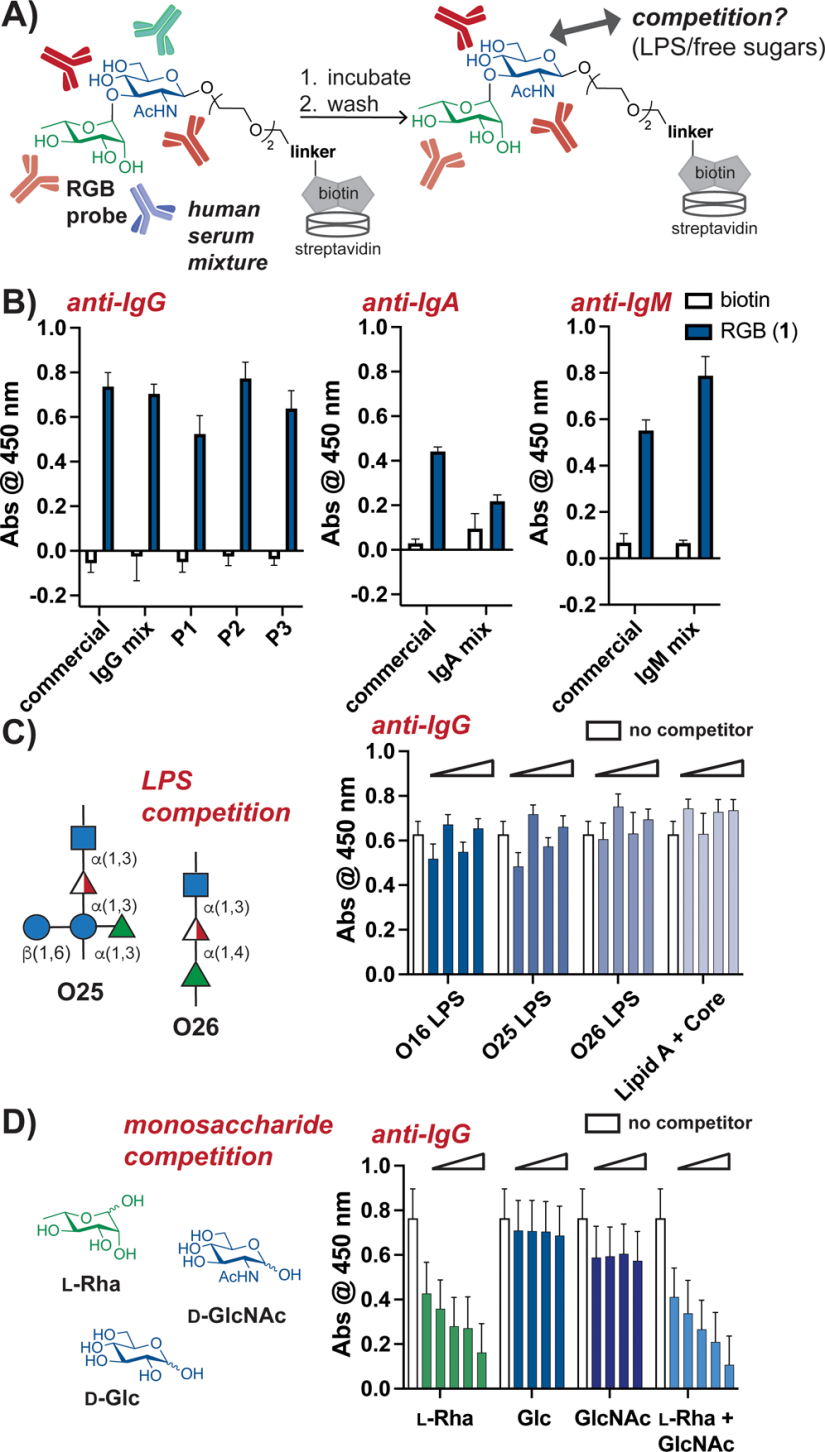
RGB probe reacts with antibodies across different human serum samples. (a) Schematic of ELISA assay for detection of bound antibodies using streptavidin-coated multiwell plates. (b) ELISA analysis of common antibody isotypes in various human serum samples indicate the presence of **RGB**-bound IgG, IgA, and IgM (commercial: commercial human serum sample; P1–P3: clinical human serum samples; IgG, IgA, and IgM mix: commercial human Ig samples containing only the indicated isotype). (C) Competition ELISA experiments using LPSs and commercial human serum suggest that bound IgG does not interact with purified LPS (0.5, 1, 2, and 4 mg/mL LPS added). Additional O-unit sequences shown. (D) Competition ELISA experiments using commercial human serum and free monosaccharides indicate that l-Rha ± GlcNAc competes in a concentration-dependent manner for binding of IgG to probe (62.5, 125, 250, and 500 mM sugar(s) added; additional 31.25 mM concentration used for l-Rha). For parts B–D, a “no probe” replicate was subtracted to account for background (*n* = 3, error bars represent standard error of the mean (SEM), Abs = absorbance).

To assess if the observed interactions between our **RGB** probe and IgG antibodies were specific, we next performed competition assays using purified LPS with different O-Ag sequences. LPSs from different strains of *E. coli* were obtained so that we could examine the effect of different sequences on the binding of human antibodies to **RGB**. As our disaccharide probe represents a fragment of O16 O-Ag, we first purified LPS from *E. coli* expressing O16 O-Ag. The resulting glycolipid was analyzed by SDS-PAGE followed by silver staining to ensure that O-Ag was present, in comparison to LPS from *E. coli* defective in O-Ag synthesis that produces only Lipid A with core oligosaccharides (Figure S2). As a comparison to the O16 glycan sequence, we purified LPS O25 and obtained commercial LPS O26.^12^ LPS O26 O-Ag has a similar polymer length as that of O16 but has a “terminal” l-Rha-containing O-unit, while LPS O25 O-Ag polymers are slightly shorter in length and consist of an O-unit with a “pendant” l-Rha residue (Figure 2C).

Upon titration of LPS O16 into wells containing RGB with bound antibodies from commercial human serum, we observed no change in the amount of bound IgG (Figure 2C). Similarly, titration of Lipid A plus core oligosaccharides, or LPS O26 or O25, did not cause displacement of the bound antibody. Further, preincubation of LPS with human serum did not lead to sequence-dependent competition with bound IgG (Figure S3). Hence, at the solubility limit of LPS, the **RGB**–antibody interaction was not disrupted in a selective manner.

As l-Rha is known to be a major antigen for human antibodies in serum, we hypothesized that free monosaccharides might better compete with interactions between **RGB** and IgG antibodies. Hence, we added increasing concentrations of l-Rha, GlcNAc, or both, using Glc as a control, to **RGB** bound to human antibodies. Interestingly, we observed that l-Rha and a mixture of l-Rha and GlcNAc competed with IgG antibodies to a similar degree in a concentration-dependent manner (Figure 2D). Glc did not compete, as expected, and added GlcNAc produced little change in the bound IgG levels. These data suggest that human antibodies primarily interacted with the l-Rha motif of our probe; however, it should be noted that free sugars do not completely release bound antibody, which indicates that the glycosidic linkage to GlcNAc may also participate in glycan–protein interactions. We hypothesized that LPS O25 and O16 could not compete for bound antibodies bound to **RGB** because effective concentrations of accessible l-Rha in heterogeneous LPS could not reach the millimolar concentrations of soluble l-Rha needed for antibody displacement.

To better understand interactions that might be made between antibodies and **RGB**, we evaluated two commercial non-humanserotyping polyclonal antibodies, anti-O16 and anti-O25, against our probe. Notably, our RGB probe did not react with any tested concentration of anti-O16 antibody, indicating that the disaccharide motif is not the primary site of recognition for these antibodies (Figure 3A). However, upon titration with the anti-O25 antibody, we observed concentration-dependent detection of **RGB**. Collectively, these data provided further support that **RGB** primarily interacted with proteins that bind to “pendant” or branched, as opposed to internal, l-Rha residues found in O-Ag (see Figures 1 and 2C).

**Figure 3:**
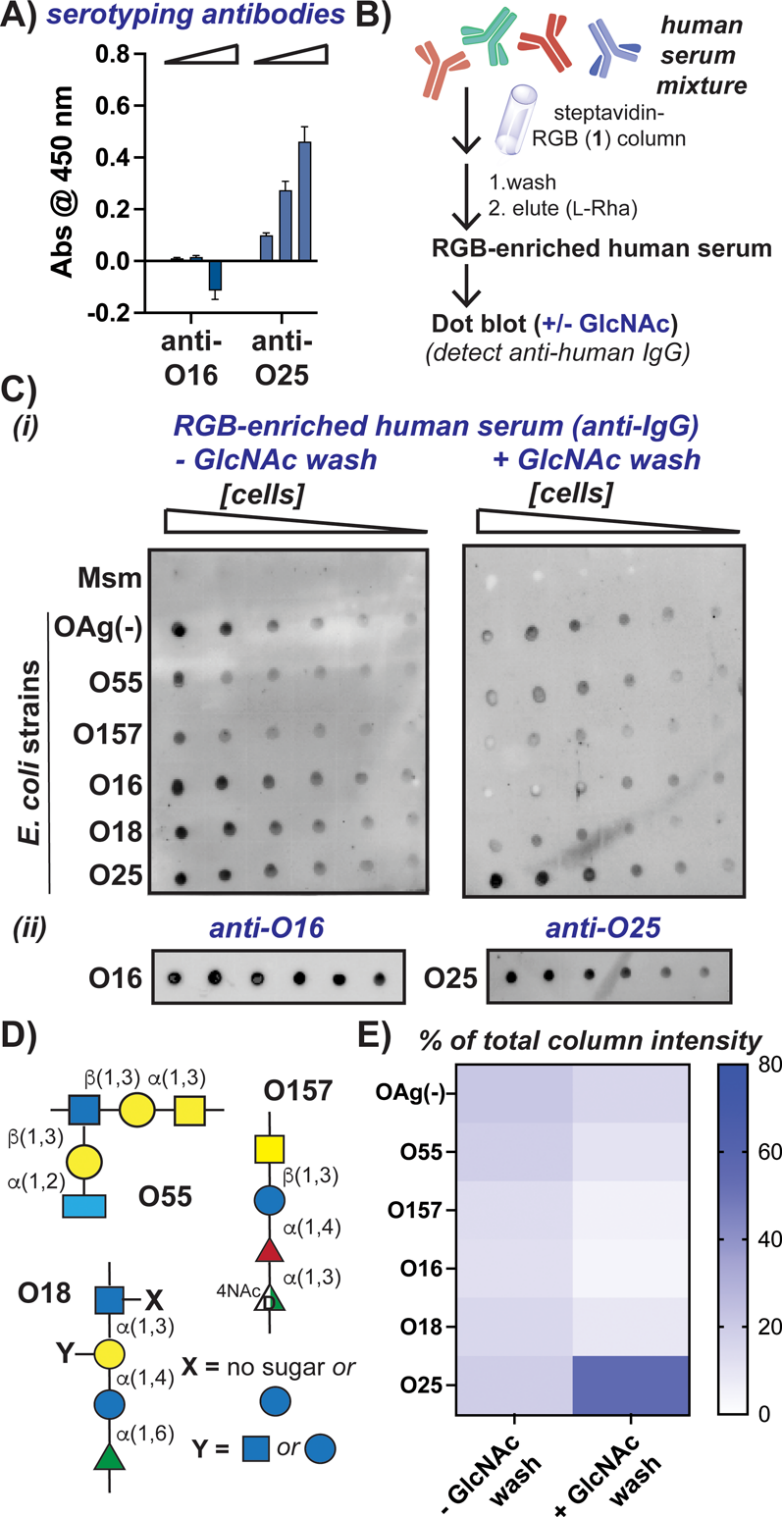
RGB-enriched serum binds to *E. coli* strain expressing branched l-Rha residues. (a) ELISA analysis of **RGB** with increasing concentrations of indicated serotyping antibodies demonstrates that anti-O25 antibodies react with **RGB**. (b) Schematic of protocol for enrichment of anti-**RGB** antibodies from commercial human sera. (C) (i) Representative dot blots of indicated bacteria strains using **RGB**-enriched serum as the primary antibody either without (left) or with (right) a GlcNAc wash step to compete with nonspecific antibodies. (ii) Dot blot analyses using commercial anti-O16 or anti-O25 antibodies. (D) Structures of O-units expressed by additional *E. coli* strains. (E) Quantification of independent replicates in each blotting condition shown in part C. Percent intensity calculated for each dot compared to the total dot intensity over the highest concentration of cells (*n* = 3, Figure S5).

With evidence that the **RGB** probe reacted with antibodies against l-Rha motifs, we last sought to isolate human anti-l-Rha antibodies to evaluate recognition of bacterial strains expressing different surface glycans. Using commercial human serum, we performed affinity chromatography with the **RGB** probe bound to streptavidin-coated beads and eluted bound antibodies with high concentrations of l-Rha (Figure 3B), as excess l-Rha competed with **RGB** bound to IgG antibodies (Figure 2D). We then performed dot blot analysis with the resulting “**RGB**-enriched” human serum using different bacterial analytes (Figure 3C,D). We examined **RGB**-enriched human serum detection of *E. coli* that lacks O16 (“OAg(−)”) and *E. coli* that expresses O16,^13^ along with *E. coli* O25, which contains a branched l-Rha residue in the repeating O-unit. *E. coli* O55 and O157 were also tested, as each produces O-Ag with different deoxysugars (l-Colitose (l-Col) in the former, *N*-acetyl rhamnosamine (RhaNAc) in the latter)^12^ (Table S3). *Mycobacterium smegmatis* (Msm) was chosen for comparison to various *E. coli* strains because mycobacteria should lack l-Rha on the cell surface, instead l-Rha is present within the cell wall beneath the “outer” mycomembrane.^17^ Analysis of blots with anti-O16 and anti-O25 serotyping antibodies validated that bacterial strains could be detected as expected using this format (Figures 3C­(ii) and S4A). Detection of bound IgG antibodies to these bacterial strains using **RGB**-enriched serum demonstrated selective detection of *E. coli* strains over Msm in a concentration-dependent manner (Figures 3C­(i), left, and S5). Surprisingly, *E. coli* strains were detected with similar intensities, even *E. coli* lacking O-Ag and the O55 serotype that lacks Rha in expressed O-Ag (Figure 3D,E). These results suggested that nonspecific detection of Gram-negative bacterial surface components may occur with the isolated antibody mixture. Notably, we observed only minor detection of *E. coli* lacking O-Ag by the secondary antibody alone (Figure S6).

Past work has shown that competition of antibody-bound glycan antigens with free ligands can be performed during dot blot analysis.^29^ As *E. coli* surfaces are rich in GlcNAc residues^11^ and our probe contains GlcNAc, we hypothesized that competition of bound antibodies with free GlcNAc might lead to enhanced surface l-Rha detection. Further, we saw that free GlcNAc competed with some **RGB**-bound IgG by ELISA analysis (Figure 2D). We first confirmed that excess GlcNAc did not displace commercial serotyping antibodies from target bacterial strains (Figure S4B). Then, upon addition of excess GlcNAc to the wash buffer following incubation of **RGB**-enriched serum with bound bacterial analytes, we observed improved detection of *E. coli* O25 compared to the other strains (Figures 3C­(i), right, and S5). The average signal intensity of *E. coli* O25 was >50% of the total intensity of comparable concentrations of other tested strains (Figure 3E, *n* = 3). When **RGB**-enriched serum was preincubated with excess l-Rha, decreased detection of *E. coli* O25 was observed, while addition of excess biotin did not affect detection (Figure S7A,B). Hence, the **RGB**-enriched serum could be used to detect l-Rha containing sequences on bacterial cells, although these antibodies exhibit a preference for branched l-Rha as opposed to “internal” l-Rha residues, the latter of which are found in O16.

Overall, our observations provide a better understanding of the recognition epitopes of anti-l-Rha antibodies from human sera. Past work on the development of defined glycan antigens derived from l-Rha-containing surface polysaccharides as vaccine candidates for *Clostridium difficile* infections has indicated that the minimum immunogenic epitope is the disaccharide fragment l-Rha­(α1,3)­Glc.^22^ Sera from mice immunized with synthetic *C. difficile* surface polysaccharides showed high levels of IgG antibodies against this disaccharide probe. However, anti-l-Rha monosaccharide antibodies did not cross-react with antibodies that reacted with larger glycan fragments,^22^ providing further evidence that l-Rha monosaccharides can be distinguished from oligosaccharides containing l-Rha by human antibodies. Additionally, the IgG response was weaker to glycan fragments that mimicked the “internal” sequence of *C. difficile* polysaccharides. In line with this observation, we found that antibodies isolated from human sera using an “internal” disaccharide fragment did not appear to recognize “internal” sugar motifs that are expressed in native O-Ags. Notably, we did not perform immunizations prior to the isolation of sera that might lead to the enrichment of anti-**RGB** antibodies; however, our goal was to understand the binding partners of naturally occurring human antibodies. While the native antigens for anti-l-Rha antibodies in humans are still unclear, as they can originate from microbial or plant sources, this work suggests that antibodies isolated by **RGB** recognize primarily free monosaccharide or pendant l-Rha residues. Accordingly, studies that identified anti-l-Rha human antibodies used l-Rha monosaccharide-containing glycoconjugates,^7–9^ and microbial glycan arrays also revealed heightened human antibody reactivity with bacterial surface polysaccharides composed of complex sequences containing l-deoxysugars.^27,30^ Hence, since **RGB**-bound antibodies were isolated by elution with l-Rha, we likely enriched human antibodies that recognize l-Rha monosaccharides over polymers. This conclusion is supported by bacterial analysis using **RGB**-bound antibodies isolated using GlcNAc, which results in nonspecific detection of strains (Figure S7C,D).

Future efforts using a broader collection of l-Rha-containing glycans may reveal more distinct recognition motifs for human anti-rare sugar antibody recruitment,^4,9^ perhaps by introducing other rare sugars in probes.^31^ Our work provides facile methods to isolate anti-l-Rha antibodies from human serum samples for the detection of relevant bacterial strains that contain terminal or branched l-Rha residues, which are prevalent in many nonmammalian surface glycan motifs, and lays the foundation for identification of natural antigens for other human anti-rare glycan antibodies.

## Supplementary Material



## Notes

Experimental procedures for syntheses; compound characterization (NMR and MS analyses) (Schemes S1–S3, Tables S1–S2); additional biochemical methods and data; list of strains used and replicate blots (Table S3, Figures S1–S7) (PDF)

## †

Department of Chemistry, Graduate School of Science, The University of Osaka, 1-1 Machikaneyamacho, Toyonaka, Osaka 560-0043, Japan

## Abbreviations

l-Rha: l-rhamnose
l-Fuc: l-fucose
l-Col: l-colitose
Glc: glucose
GlcNAc: *N*-acetyl glucosamine
Man: mannose
Gal: galactose
Gal*f*: galactofuranose
GalNAc: *N*-acetyl galactosamine
MurNAc: *N*-acetyl-muramic acid
GalA: galacturonic acid
l-FucNAc: *N*-acetyl-l-fucosamine
RhaNAc: *N*-acetyl rhamnosamine
O-Ag: O-antigen
LPS: lipopolysaccharide
ELISA: enzyme-linked immunosorbent assay
