# Supplementary material for: Microbial Surface Glycan Probe Isolates Anti‑l‑Rhamnose Antibodies from Human Serum for Bacterial Detection
Source: ACS Infect Dis. 2025 Nov 25;11(12):3414–9. doi: 10.1021/acsinfecdis.5c00757 (PMC12706776; doi:10.1021/acsinfecdis.5c00757)

**Supporting Information**

# Microbial surface glycan probe isolates anti-L-Rhamnose antibodies from human serum for bacterial detection

Hersa Milawati, Mia Sheshova, Joanna Joo, Tania J. Lupoli*

Department of Chemistry, New York University, New York, NY 10003

*email: tjl229@nyu.edu

## Experimental Synthetic Procedures and Data

**General Information for synthetic compounds.** All solvents were purchased from Fisher Scientific or Sigma Aldrich. All chemicals were purchased from Fisher Scientific, Sigma Aldrich, Alfa Aesar, TCI chemicals, Chem-Impex or Biosynth, and used without further purification. All reactions were performed under nitrogen atmosphere. Column chromatography was performed on silica gel 60, 230-400 mesh from Fisher Scientific. Analytical thin layer chromatography was performed on silica gel 60 F254 aluminum plates, which were visualized under UV (254 nm) and by staining with ceric ammonium molybdate (CAM) followed by brief heating. NMR spectra were obtained using a Bruker Avance III HD 400 NMR Spectrometer at 400 MHz for $^{1}H$ NMR and 101 MHz for $^{13}C$ NMR or Bruker AV4-500 NMR Spectrometer at 500 MHz for $^{1}H$ NMR and 125 MHz for $^{13}C$ NMR. Chemical shifts of $^{1}H$ and $^{13}C$ NMR were represented as δ-values relative to the internal standard tetramethylsilane (TMS) with references to the solvent peak ($CDCl_3$: $^{1}H$ δ 7.26 ppm, $^{13}C$ δ 77.0 ppm, $CD_3OD$ $^{1}H$ δ 3.30 ppm, $^{13}C$ δ 49.3 ppm, $D^2O$: $^{1}H$ δ 4.80 ppm), followed by processing by MestReNova (authorized to NYU). Coupling constants (*J*) were reported in Hertz (Hz). Multiplicities abbreviations: s = singlet, d = doublet, t = triplet, q = quartet, m = multiplet, br = broad. High resolution mass spectrometry analyses were acquired on an Agilent 6224 Accurate-Mass Time-of-Flight LC/MS (LC-TOF) spectrometer with an electrospray (ESI) ionization source equipped with autosampler. The following common sugar abbreviations are used: glucose (Glc) and *N*-acetyl-glucosamine (GlcNAc).

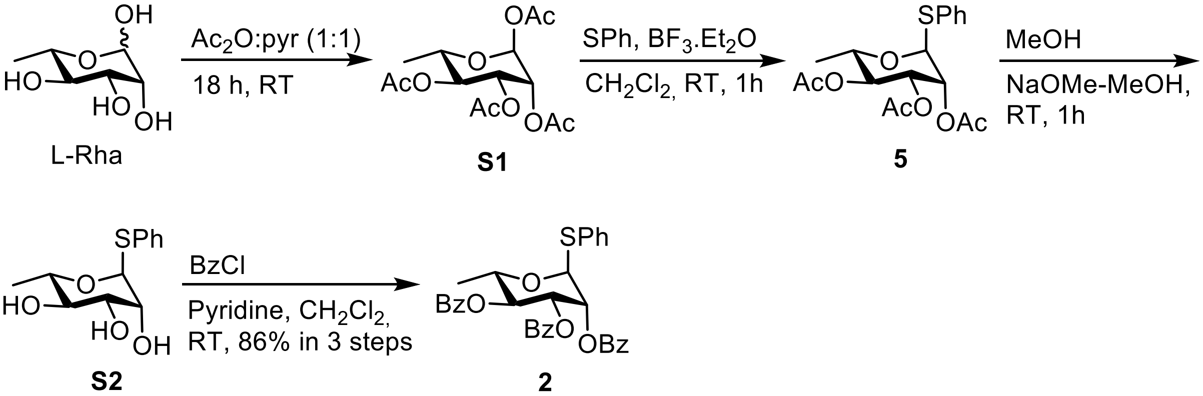


**Scheme S1.** Synthesis of **2** ($Ac_2O$ = acetic anhydride, pyr = pyridine, SPh = thiophenol, Ph = phenyl, $BF_3{\cdot}Et_2O$ = boron trifluoride etherate, NaOMe = sodium methoxide, MeOH = methanol, BzCl = benzoyl chloride, RT = room temperature = 25 $^{o}C$)

### Compound S1

In a round-bottom flask, to a solution of L-rhamnose (L-Rha) (5 g, 30 mmol) in anhydrous pyridine (16 mL) was added acetic anhydride ($Ac_2O$, 16 mL) at RT (25 $^{o}C$). After being stirred for 18 hours at RT under $N_2$ atmosphere and confirming the completion of the reaction by TLC (Hexane:EtOAc = 1:1, Rf = 0.6), the reaction mixture was concentrated in vacuo and co-evaporated with toluene six times to afford crude product **S1**[1] as yellow oil. The crude product was directly used for the next reaction without further purification.

**Compound 5**

To a round-bottom flask, the crude yellow oil **S1** (30 mmol) in anhydrous $CH_2Cl_2$ (37.5 mL) was added thiophenol (SPh, 3.7 mL, 36 mmol) followed by cooling to 0 °C. After being stirred for 10 minutes, boron trifluoride diethyl etherate ($BF_3.Et_2O$, 4.4 mL, 36 mmol) was slowly added into the mixture at 0 °C under $N_2$ atmosphere. After the reaction was stirred for 1 hour at RT (25 °C), and completion of the reaction was confirmed by TLC (Hexane:EtOAc = 1:1, Rf = 0.8), the reaction mixture was quenched with saturated $NaHCO_{3(aq)}$, followed by extraction with $CH_2Cl_2$ three times. The organic layer was dried over $Na_2SO_4$, filtered and concentrated in vacuo to afford crude product **5**[1] as yellowish oil. The crude product was directly used for the next reaction without further purification.

**Compound S2**

In a round-bottom flask, to a solution of crude yellowish oil **5** (5.1 g, 13.3 mmol) in anhydrous MeOH (95 mL) was added 0.5 M of NaOMe in MeOH (52 mL, 26.6 mmol) slowly. After stirring for t = 1 hour at RT (25 °C) under $N_2$ atmosphere and confirming completion of the reaction by TLC ($CHCl_3$:MeOH = 9:1, Rf = 0.4), the reaction mixture was quenched using Amberlite IRC-120(H) until the pH reached 7.0, followed by filtration. The reaction mixture was concentrated in vacuo to afford crude product **S2**[1] as a yellowish oil, which was used for the next reaction without further purification.

**Compound 2**

For the synthesis of **2**, a reported method[2] was slightly adjusted. In a round-bottom flask, to a solution of crude yellowish oil **S2** (13.3 mmol) in anhydrous $CH_2Cl_2$ (133 mL) were added pyridine (18.2 mL, 226 mmol) followed by benzoyl chloride (BzCl, 15.4 mL, 133 mmol) slowly at 0 °C. After being stirred for t = 18 hours under $N_2$ atmosphere, and confirming the completion of the reaction by TLC (Hexane:EtOAc = 1:1, Rf = 0.9, α:β ~ 9:1), the reaction mixture was quenched using $NaHCO_{3(aq)}$, followed by extraction with $CH_2Cl_2$ three times, and then concentrated in vacuo. The residue was purified by silica-gel column chromatography (Hexane:EtOAc = 10:0 to 8:2, 5% gradient) to yield compound **2** (6.4 g, 86%) as white solid; the anomeric configuration was confirmed by NMR analysis (**Table S1**).

$^1$H-NMR (400 MHz, $CDCl_3$): δ (ppm) = 8.00 (m, 2H, H aromatic), 7.92 (m, 2H, H aromatic), 7.76 (m, 2H, H aromatic), 7.51-7.19 (m, 14H, H aromatic), 5.85 (dd, *J* = 1.6; 3.1 Hz, 1H, H-2), 5.73 (dd, *J* = 3.1; 10.0 Hz, 1H, H-3), 5.68 (t, *J* = 10.0 Hz, 1H, H-4), 5.62 (d, *J* = 1.6, 1H, H-1), 4.57 (m, 1H, H-5), 1.31 (d, *J* = 6.2 Hz, 3H, H-6).

$^{13}$C{1H} NMR (101 MHz, $CDCl_3$): δ (ppm) = 172.6, 165.9, 165.6, 133.8 (X2), 133.6, 133.5, 133.3, 133.3, 132.0, 130.3 (X3), 129.9, 129.8, 129.8, 129.4, 129.4, 129.3, 129.2, 129.0, 128.7, 128.5 (X3), 128.4, 128.0, 85.9, 72.4, 71.9, 70.4, 68.2, 17.6.

HRMS (LC-TOF-MS) *m/z* calculated for $C_{33}H_{28}O_7SNa$ $[M+Na]^+$ 591.1453, found 591.1444 (mass error: -1.52 ppm)

**Table S1.** List of chemical shifts (δ) and coupling constants (*J*) of relevant anomeric protons.

| Compound No. | Chemical Shift (H-1, ppm) | Chemical Shift (C-1), ppm | Coupling Constant, $^3J_{H1,H2}$ (Hz) |
|---|---|---|---|
| **2** | 5.62 | 85.9 | 1.6 |
| **3** | 5.22 | 99.0 | 8.4 |
| **6** | 5.25; 4.74 | 99.0; 97.1 | 8.4; 1.6 |
| **7** | 5.10; 5.01 | 97.4; 100.7 | 1.7; 8.3 |
| **8** | 4.73; 4.38 | 101.8; 100.9 | Not observable; 8.5 |
| **1** | 4.73; 4.40 | 101.0; 100.1 | 1.8; not observable |

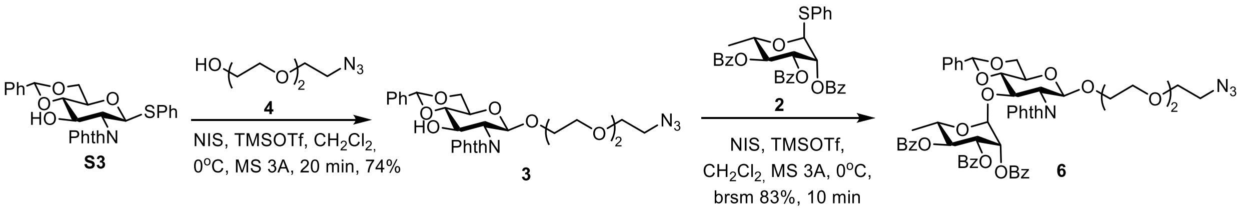


**Scheme S2.** Synthesis of disaccharide **6** (NIS = *N*-iodosuccinimide, TMSOTf = trimethylsilyl trifluoromethanesulfonate, MS = molecular sieves, Phth = phthaloyl, brsm = based on recovery starting material)

**Compound 3**

In a round-bottom flask, to a solution of phenyl 4,6-*O*-benzylidene-2-deoxy-2-phthalimido-β-D-thioglucopyranoside **S3** (Biosynth, 200 mg, 0.41 mmol), **4**[3] (59.6 mg, 0.34 mmol), *N*-iodosuccinimide (NIS, 114.7 mg, 0.51 mmol) and activated MS (3 Å) in $CH_2Cl_2$ (3.4 mL) was added trimethylsilyl trifluoromethanesulfonate (TMSOTf, 6.1 µL, 0.034 mmol) at 0 °C. After being stirred for t = 20 minutes at 0 °C under $N_2$ atmosphere, and confirming the progress of the reaction by TLC (hexane:EtOAc = 1:1, Rf = 0.3), the reaction mixture was then diluted with $CH_2Cl_2$ and quenched with TEA until the pH = 7.0, followed by addition of saturated aq. $Na_2S_2O_3$ and subsequent extraction with $NaHCO_3$ and $CH_2Cl_2$. The organic layer was dried over $Na_2SO_4$, filtered and concentrated in vacuo. The residue was purified by silica-gel column chromatography (Hexane:EtOAc = 10:0 to 7:3) to give compound **3** (130.7 mg, 70%) as colorless oil. The NMR spectra was identical to the reported data for the same compound[4]. Based on TLC and NMR analysis (**Table S1**), only the β-anomeric product was obtained. The byproduct succinimide was also detected.

$^1$H-NMR (400 MHz, $CDCl_3$): δ (ppm) = 7.77 (m, 2H, H aromatic), 7.65 (m, 2H, H aromatic), 7.42 (m, 2H, H aromatic), 7.30 (m, 3H, H aromatic), 5.49 (s, 1H, H benzylidene), 5.22 (d, *J* = 8.4 Hz, 1H, H-1), 4.56 (dd, *J* = 8.4; 10.4 Hz, 1H, H-3), 4.29 (dd, *J* = 4.4; 10.4 Hz, 1H), 4.16 (dd, *J* = 8.4; 10.4 Hz, 1H, H-2), 3.80 (m, 1H, H-5), 3.75 (t, *J* = 9.8 Hz, 1H), 3.56 (m, 3H, H-6, H linker), 3.41 (m, 4H, H linker), 3.26 (m, 6H, H linker).

$^{13}C\{1H\}$ NMR (101 MHz, $CDCl_3$): δ (ppm) = 168.4, 168.2, 137.1, 134.2 (X2), 131.8, 129.4, 128.5 (X3), 126.4 (X3), 123.5, 102.0, 99.1, 82.3, 70.6, 70.5, 70.2, 70.0, 69.3, 68.8, 68.6, 66.2, 56.7, 50.7

HRMS (LC-TOF-MS) *m/z* calculated for $C_{27}H_{40}N_4O_9K_2$ $[M+2K]^{+2}$ 321.1029, found 321.1034 (mass error: 1.53 ppm)

**Compound 6**

The conditions for the reaction were optimized as shown in **Table S2**. Entry 4 was used for isolation of product. In a round-bottom flask, to a solution of **3** (82.2 mg, 0.14 mmol), **2** (126.1 mg, 0.22 mmol), *N*-iodosuccinimide (NIS, 40 mg, 0.18 mmol) and activated MS (3Å) in $CH_2Cl_2$ (2.9 mL) were added, along with trimethylsilyl trifluoromethanesulfonate (TMSOTf, 13.4 μL, 0.074 mmol) at 0 °C. After being stirred for t = 10 minutes at 0 °C under $N_2$ atmosphere and confirming the progress of the reaction by TLC (hexane:EtOAc = 1:1, Rf = 0.4), the reaction mixture was then diluted with $CH_2Cl_2$ and quenched with TEA until the pH = 7.0, followed by addition of saturated aq. $Na_2S_2O_3$ and subsequent extraction with $NaHCO_3$ and $CH_2Cl_2$. The organic layer was dried over $Na_2SO_4$, filtered and concentrated in vacuo. The residue was purified by silica-gel column chromatography (Toluene:EtOAc = 10:0 to 8:2, 10% gradient) to give compound **6** (90.9 mg, 83% brsm) as a colorless oil.

**Table S2.** Glycosylation optimization between rhamnosyl donor **2** and glucosyl acceptor **3**

| Ent. | 3 (mg) | 3 (eq.) | 2 (eq.) | TMSOTf (eq.) | Time (min) | Isolated yield (%) |
|---|---|---|---|---|---|---|
| 1 | 40.6 | 1 | 1.2 | 0.3 | 10 | 60 |
| 2 | 65 | 1 | 1.2 | 0.3 | 35 | 50 |
| 3 | 65 | 1 | 1.5+0.15 | 0.3+0.3 | 35 | b.r.s.m. 76* |
| 4 | 82.2 | 1 | 1.5 | 0.5 | 10 | b.r.s.m. 83* |

*purified by toluene/EtOAc

$^1H$-NMR (400 MHz, $CDCl_3$): δ (ppm) = 7.78 (m, 2H, H aromatic), 7.72-7.66 (m, 4H, H aromatic), 7.58 (m, 2H, H aromatic), 7.43 (m, 4H, H aromatic), 7.29 (m, 6H, H aromatic), 7.13 (m, 6H, H aromatic), 5.68 (dd, *J* = 3.5; 10.1 Hz, 1H, H-3 Rha), 5.59 (s, 1H, H benzylidene), 5.35 (t, *J* = 10.1 Hz, 1H, H-4 Rha), 5.25 (d, *J* = 8.4 Hz, 1H, H-1 Glc), 5.04 (dd, *J* = 1.5; 3.5 Hz, 1H, H-2 Rha), 4.74 (d, *J* = 1.6 Hz, 1H, H-1 Rha), 4.72 (dd, *J* = 9.1; 10.4 Hz, 1H, H-3 Glc), 4.36 (m, 2H), 4.26 (m, 1H, H-5 Glc), 3.84 (m, 2H), 3.78 (t, *J* = 9.1 Hz, 1H, H-2 Glc), 3.62 (m, 2H, H linker), 3.43 (m, 4H, H linker), 3.27 (m, 6H, H linker), 0.63 (d, *J* = 6.1 Hz, 3H, C$\underline{H_3}$-6 Rha).

$^{13}C\{1H\}$ NMR (101 MHz, $CDCl_3$): δ (ppm) = 171.2 (X2), 165.7, 165.3, 165.0, 137.2, 133.9, 133.3 (X2), 133.1, 129.7 (X4), 129.6 (X2), 129.5, 129.3, 129.3, 129.2, 128.4 (X3), 128.4

(X3), 128.3 (X3), 128.2 (X3), 126.4 (X3), 102.2, 99.1, 97.2, 80.5, 73.9, 71.8, 71.5, 70.6, 70.5, 70.2, 70.2, 69.3, 69.3, 68.9, 66.7, 66.5, 56.5, 50.7, 16.7

HSQC (values listed in ppm) = $\delta_H$ 5.59 $\delta_C$ 102.2 (black line, H benzylidene), $\delta_H$ 5.25 $\delta_C$ 99.1 (red line, H-1 Glc), $\delta_H$ 4.74 $\delta_C$ 97.2 (blue line, H-1 Rha)

HRMS (LC-TOF-MS) *m/z* calculated for $C_{54}H_{52}N_4O_{16}K_2$ $[M+2K]^{+2}$ 545.1321, found 545.1351 (mass error: 5.64 ppm)

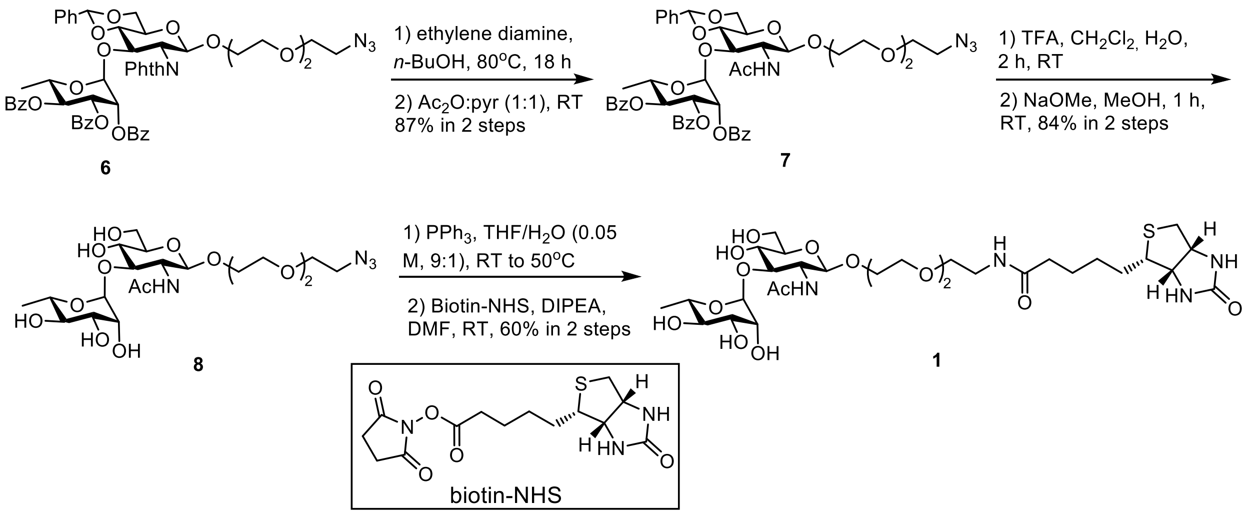


**Scheme S3.** Global deprotection and biotin coupling to obtain **1** (*n*-BuOH = *n*-butanol, $Ac_2O$ = acetic anhydride, pyr = pyridine, TFA = trifluoroacetic acid, NaOMe = sodium methoxide, MeOH = methanol, $PPh_3$ = triphenylphosphine, THF = tetrahydrofuran, DIPEA = diisopropylethylamine, DMF = dimethylformamide, RT = room temperature = 25 °C)

## Compound 7

For compound **7**, a reported method[5] was slightly adjusted. In a round-bottom flask, to a solution of **6** (90.9 mg, 0.09 mmol) in *n*-butanol (3.0 mL) were added ethylene diamine (44.6 µL, 0.67 mmol) at RT (25 °C). After being stirred for t = 18 hours at 80 °C under $N_2$ atmosphere and confirming the progress of the reaction by TLC (hexane:EtOAc = 1:1, Rf = 0.5). The reaction mixture was co-evaporated using toluene in vacuo to afford crude product as a yellowish oil. The yellowish crude oil was then dissolved in pyridine (1.5 mL) followed by the addition of acetic anhydride ($Ac_2O$, 1.5 mL). After being stirred for t = 18 hours at RT under $N_2$ atmosphere and confirming the progress of the reaction by TLC (Hexane:EtOAc = 3:2, Rf = 0.3), the reaction mixture was co-evaporated using toluene three times. The residue was purified by silica-gel column chromatography (Hexane:EtOAc = 6:4 to 4:6, 5% gradient) to give compound **7** (72.3 mg, 87%) as a yellowish oil.

$^1$H-NMR (400 MHz, $CDCl_3$): δ (ppm) = 7.99 (m, 2H, H aromatic), 7.78 (m, 4H, H aromatic), 7.54-7.12 (m, 14H, H aromatic), 6.24 (d, *J* = 7.8 Hz, 1H, -N<u>H</u>), 5.72 (dd, *J* = 3.4; 10.1 Hz, 1H, H-3 Rha), 5.54 (s, 1H, H benzylidene), 5.47 (t, *J* = 10.1 Hz, 1H, H-4 Rha), 5.42 (dd, *J* = 1.7; 3.4 Hz, 1H, H-2 Rha), 5.10 (d, *J* = 1.7 Hz, 1H, H-1 Rha), 5.01 (d, *J* = 8.3 Hz, 1H, H-

1 Glc), 4.42 (t, *J* = 9.5 Hz, 1H, H-2 Glc), 4.31 (m, 2H), 3.88 (m, 1H), 3.73 (m, 2H), 3.64-3.49 (m, 11H), 3.34 (m, 2H, H linker), 2.01 (s, 3H, $\underline{CH_3}$-C=O), 0.68 (d, *J* = 6.2 Hz, 3H, $\underline{CH_3}$-6 Rha)

$^{13}$C{1H} NMR (101 MHz, $CDCl_3$): δ (ppm) = 171.7, 165.8, 165.8, 165.7, 137.3, 133.6, 133.3, 133.2, 129.9 (X2), 129.8 (X2), 129.8 (X2), 129.6, 129.4, 129.3, 128.7 (X2), 128.5 (X2), 128.4 (X2), 128.3 (X2), 126.5 (X3), 102.2, 100.8, 97.6, 80.4, 75.5, 71.9, 71.6, 71.0, 70.8, 70.7, 70.0, 69.9, 69.2, 69.0, 66.6, 66.4, 58.5, 50.7, 23.6, 16.8

HSQC = $\delta_H$ 5.54 $\delta_C$ 102.2 (black line, H benzylidene), $\delta_H$ 5.10 $\delta_C$ 97.6 (red line, H-1 Rha), $\delta_H$ 5.01 $\delta_C$ 100.8 (blue line, H-1 Glc)

HRMS (LC-TOF-MS) *m/z* calculated for $C_{48}H_{52}N_4O_{15}Na$ $[M+Na]^+$ 947.3327, found 947.3236 (mass error: -9.60 ppm)

**Compound 8**

In a round-bottom flask, to a solution of **7** (57.7 mg, 0.063 mmol) in $CH_2Cl_2$ (1.9 mL) and $H_2O$ (190 uL) was added trifluoroacetic acid (TFA, 337.4 uL, 4.41 mmol) at RT (25 $^oC$). After being stirred for t = 1 hour at RT under $N_2$ atmosphere and confirming the completion of the reaction by TLC (Chloroform:MeOH = 49:1, Rf = 0.5),the reaction mixture was co-evaporated using toluene in vacuo to afford crude product as a colorless oil. The colorless oil was then dissolved in dry MeOH (630 µL) followed by the addition of sodium methoxide (NaOMe, 252 µL, 0.12 mmol) at RT. After being stirred for t = 1 hour at RT under $N_2$ atmosphere and confirming the progress of the reaction by TLC (chloroform:MeOH = 4:1, Rf = 0.3), the reaction was quenched using Amberlite IRC-120(H) until the pH = 7.0, followed by filtration. The reaction mixture was then concentrated in vacuo. The residue was purified by silica-gel column chromatography (chloroform:MeOH = 10:0 to 8:2, 5% gradient) to give compound **8** (27.9 mg, 84%).

$^1$H-NMR (500 MHz, $CD_3OD$): δ (ppm) = 4.73 (s, 1H, H-1 Rha), 4.38 (d, *J* = 8.5 Hz, 1H, H-1 Glc), 3.86 (m, 1H), 3.77 (m, 1H), 3.66 (m, 2H), 3.56 (m, 12H), 3.47 (t, *J* = 7.7 Hz, 1H), 3.28 (m, 4H), 3.21 (m, 1H), 1.89 (s, 3H, $\underline{CH_3}$-C=O), 1.13 (d, *J* = 6.2 Hz, 3H, $\underline{CH_3}$-6 Rha)

$^{13}$C{1H} NMR (125 MHz, $CD_3OD$): δ (ppm) = 173.5, 103.2, 102.3, 82.7, 77.9, 73.7, 72.6, 72.1, 71.6, 71.5, 71.5, 71.0, 70.6, 70.3, 69.9, 62.6, 56.7, 51.7, 23.0, 17.8

HSQC = $\delta_H$ 4.73 $\delta_C$ 103.2 (black line, H-1 Rha), $\delta_H$ 4.38 $\delta_C$ 102.3 (blue line, H-1 Glc)

HRMS (LC-TOF-MS) *m/z* calculated for $C_{20}H_{36}N_4O_{12}Na$ $[M+Na]^+$ 547.2227, found 547.2220 (mass error: -1.27 ppm)

**Compound 1**

In a round-bottom flask, to a solution of **8** (9.3 mg, 0.018 mmol) in THF (324 µL) and $H_2O$ (36 µL) were added triphenylphosphine ($PPh_3$, 4.8 mg, 0.018 mmol) at RT (25 $^oC$). After being stirred for t = 18 hours at 50 $^oC$ under $N_2$ atmosphere, the complete consumption of **8** was confirmed by TLC (chloroform:MeOH = 3:1) and product formation was indicated by HR-MS (*m/z* calculated for $C_{20}H_{38}N_2O_{12}Na$ $[M+Na]^+$ 521.2322, found 521.2290), the

reaction mixture was concentrated in vacuo to afford crude product. The crude product was then dissolved DMF (360 μL) followed by the addition of DIPEA (4.7 μL, 0.027 mmol) and (+)-biotin *N*-hydroxysuccinimide ester (biotin-NHS) (ChemImpex, 7.3 mg, 0.021 mmol) at RT. After being stirred for t = 1 hour at RT under $N_2$ atmosphere and confirming the completion of the reaction by TLC (chloroform:MeOH = 3:1, Rf = 0.1), the reaction mixture was co-evaporated using toluene in vacuo. The residue was purified by silica-gel column chromatography (chloroform:MeOH = 10:0 to 3:1, 20% gradient) to give the final compound **1** (7.5 mg, 60%).

$^{1}$H-NMR (500 MHz, $CD_3OD$): δ (ppm) = 4.73 (d, *J* = 1.8 Hz, 1H, H-1 Rha), 4.49 (s, 4H, 4X N$\underline{H}$), 4.40 (m, 2H, H-1 Glc, biotin C$\underline{H}$), 4.21 (dd, *J* = 4.4; 7.8 Hz, 1H, biotin C$\underline{H}$), 3.85 (m, 2H), 3.77 (dd, *J* = 2.2; 12 Hz, 1H), 3.66 (m, 2H), 3.60 (m, 2H), 3.55- 3.42 (m, 11H), 3.26 (m, 4H), 3.11 (m, 1H, biotin -S-C$\underline{H}$), 2.82 (dd, *J* = 4.9; 12.7 Hz, 1H, biotin C$\underline{H_2}$), 2.59 (d, *J* = 12.7 Hz, 1H, biotin C$\underline{H_2}$), 2.13 (t, *J* = 7.4 Hz, 2H, biotin -NH-CO-C$\underline{H_2}$), 1.89 (s, 3H, C$\underline{H_3}$-C=O), 1.68-1.31 (m, 6H, biotin linker 3X C$\underline{H_2}$), 1.13 (d, *J* = 6.1 Hz, 3H, C$\underline{H_3}$-Rha)

$^{13}$C{1H} NMR (125 MHz, $CD_3OD$): δ (ppm) = 176.2, 173.4, 166.1, 103.3, 102.3, 83.8, 78.0, 73.7, 72.6, 72.1, 71.6, 71.5, 71.3, 70.7, 70.6, 70.4, 70.0, 63.4, 62.7, 61.6, 57.0, 56.7, 41.0, 40.3, 36.7, 29.7, 29.5, 26.8, 23.0, 17.8

HSQC = $\delta_H$ 4.73 $\delta_C$ 103.3 (black line, H-1 Rha), $\delta_H$ 4.40 $\delta_C$ 102.3 (blue line, H-1 Glc)

HMBC = $\delta_H$ 4.40 $\delta_C$ 166.1 (black line, Biotin CH), $\delta_H$ 4.21 $\delta_C$ 166.1 (red line, Biotin CH)

HRMS (LC-TOF-MS) *m/z* calculated for $C_{30}H_{52}N_4O_{14}SNa$ $[M+Na]^+$ 747.3098, found 747.3108 (mass error: 1.33 ppm)

**Cell-based and Biochemical Procedures and Data**

**General Methods.** Reagents were purchased from Sigma-Aldrich unless otherwise noted. Phosphate buffered saline (PBS) (10X, magnesium chloride and calcium chloride free, pH 7.4) was from Gibco and was diluted in double distilled water (dd$H_2O$) to 1X. GraphPad Prism software 9.0 was used for data analysis and plotting. Gel band quantification was performed in Image Lab (Bio-Rad, band percent calculation) after image files were saved using ImageJ (NIH). *E. coli* strains were grown in Luria Bertani (LB) broth, and *M. smegmatis* was grow in Middlebrook 7H9 medium (BD Difco) supplemented with 2% glycerol, 0.05% Tween-80, 0.5% bovine serum albumin (BSA, Roche), 0.2% dextrose, and 0.085% NaCl (called 7H9 complete/tween). Trisbuffered saline (TBS, 50 mM tris(hydroxymethyl)aminomethane (Tris), pH 7.5, 150 mM NaCl) was made in house.

Both commercial and human serum samples were used in this study. Commercial human serum was from human male AB plasma (Sigma-Aldrich, H4522). Purified human isotypes isolated from human serum samples were from commercial sources: IgG from human serum (Sigma-Aldrich I4506), IgA from human serum (Sigma-Aldrich, I4036) or IgM from human serum (Sigma-Aldrich, I8260). Three de-identified clinical human serum samples were obtained from NYU Langone Center for Biospecimen Research and Development (samples P.00251808 (HS.L.1), P.00055233 (HS.L.2), P.00018205 (HS.L.3). All human serum samples were obtained as frozen stocks. Once received, they were aliquoted (50 µL for clinical samples and 250 µL for commercial samples) and frozen at -80 °C. Aliquots were only thawed once for immediate use in experiments and unused remaining samples were typically discarded.

**Bacterial culture preparation for lipopolysaccharide (LPS) extraction**. All bacterial work was performed in a BSL2 biosafety hood. The overnight cultures were prepared by inoculating the frozen cell stock in 3 mL of LB (IBI Scientific) and incubating overnight at 30 °C with 150 rpm shaking. After overnight incubation, the cells were diluted to 1:30 in LB and grown to log phase to optical density at 600 nm (OD600) of 0.6 at 37 °C with 200 rpm shaking. The cells were further diluted to 1:30 in LB with supplement of 0.5% DMSO. The cells were grown to mid-log phase at 37 °C with 200 rpm shaking and were normalized to OD600 of 0.5 prior to extraction.

**LPS extraction with silver staining and immunoblot.** LPS extraction was achieved by adapted published protocols.[6] The normalized cultures (1.5 mL) were centrifuged at 15,871 x g for 2 min to obtain pellets. The supernatant was discarded, and the pellet was resuspended with 200 µL of 1x sodium dodecyl sulfate (SDS) buffer. 1x SDS buffer was prepared by diluting 2x SDS buffer (4% β-mercaptoethanol (BME), 4% (w/v) SDS and 20% glycerol in 0.1 M Tris-HCl pH 6.8, 0.01% bromophenol blue) 1:1 in d$H_2O$. The resuspended samples were boiled at 100 °C for 15 min. Then, the boiled samples were cooled down to RT. Into each sample, 10 µL of 5 mg/mL DNase I and 5 µL of 10 mg/mL RNase were added and the samples were incubated at 37 °C for 30 min. Then, 5 µL of 20 mg/mL Proteinase K was added to each sample and the samples were incubated at 60 °C overnight or for 3 hrs. To each sample, 200 µL of cold Tris-saturated phenol was

added for extraction. The samples were vortexed and incubated at 65 °C for 15 min, vortexing occasionally. After cooling down to room temperature, 1 mL of diethyl ether was added. The samples were vortexed and centrifuged at 15,871 x g for 10 min. The bottom blue layers were collected, and the samples were re-extracted. To each of extracted LPS samples, an equal volume of 2x SDS buffer was added. The samples were loaded onto 15% gels and analyzed by SDS-PAGE (polyacrylamide gel electrophoresis) at 180 V for 40 min in 1x SDS-PAGE running buffer (25 mM Tris, 191 mM glycine, 0.1% (w/v) SDS). The gel was rocked overnight in fixing solution (55% dd$H_2O$, 40% EtOH, 5% acetic acid, 200 mL), which was replaced the following day with fixing solution supplemented with 0.7% periodic acid (5 min). The gel was washed 3x in dd$H_2O$ (200 mL, 15 min) and treated with freshly prepared staining solution (0.019 M NaOH, concentrated 0.193 M $NH_4OH$, 0.67% $AgNO_3$, 150 mL). The gel was then washed 3x in dd$H_2O$ (200 mL, 15 min) and developed with freshly prepared developing solution (0.26 mmol citric acid, 0.0185% (v/v) formaldehyde, 500 mL) and was allowed to stain until the desired intensity had been achieved.

For western blot analysis, samples were analyzed by SDS-PAGE as described above for silver staining. Immunoblotting was performed by transfer onto polyvinylidene difluoride membrane (PVDF) at 25 V for 30 min and incubated with blocking buffer (2.5% (w/v) milk fat in 1x TBS supplemented with 0.1% Tween (TBS-T)) for 1 hr at 25 °C. Primary antibody was incubated overnight at 4 °C in blocking buffer with the following dilution: anti-O16 or anti-O25, 1:500, (SSI Dianostica, catalog numbers 85012 and 85022, respectively). After washing with TBS-T (1 hr at room temperature), secondary antibody (anti-Rabbit HRP, 1:3000, R&D Systems) in blocking buffer was then incubated with the membrane overnight at 4 °C prior to washing with TBS-T (1 hr at room temperature) and imaging with Clarity MaxTM Western ECL Substrate (Bio-Rad).

**LPS extraction to obtain pure LPS for competition assays.** LPS extraction was achieved by adapted published protocols.[7] An overnight culture (100 mL) was used of relevant strains (*E. coli* MG1655 *wbbL*- (Lipid A + core), *E. coli* MG1655 *wbbL*+ O16,[8] *E. coli* O25:H4), which were washed twice with PBS supplemented with 0.15 mM $CaCl_2$ and 0.5 mM $MgCl_2$. Cells were sonicated for 10 min (30 sec on; 30 sec off, Amplitude 50%, Fisherbrand 120 Sonic Dismembrator). Lysed cells were incubated at 65 °C for one hour with Proteinase K (100 mg/mL, Thermo Scientific). Lysed cells were then supplemented with RNase A (40 µg/mL), DNase I (20 µg/mL), $MgSO_4$ (1 µL/mL), and chloroform (4 µL/mL) and incubated at 37 °C overnight. The next day, cells were treated with Tris-saturated phenol (Thermofisher) pre-heated to 65 °C and mixed vigorously for 15 min. The mixture was cooled to 0 °C for 15 min and subjected to centrifugation (1857 x g, 10 min, 4 °C, Beckman Coulter Allegra X-15R). The aqueous phase was removed, and the organic phase was extracted with dd$H_2O$ (2 mL) preheated to 65 °C. Cells were cooled and centrifuged as described above. The combined aqueous phases were diluted with 35 mL of 95% ethanol and treated with sodium acetate (final concentration 0.5 M) and cooled overnight (-20 °C). The sample was centrifuged (1857 x g, 10 min, 4 °C), the pellet was resuspended in dd$H_2O$ and dialyzed against dd$H_2O$ for 4 days. The resulting product was lyophilized to yield a white powder and stored at 4 °C indefinitely.

**Enzyme-Linked Immunosorbent Assay (ELISA) for analysis of glycan detection by antibodies without and with competitors.** Protocol was adjusted based on the manufacturer's instructions. Multiwell streptavidin-coated plates (Acro Biosystem, SP-11-5 plates) were initially washed with 200 µL of buffer (1X TBS supplemented with 0.1% Tween-20 (TBS-T)) three times every 5 minutes. To the wells were then added 50 µL of 100 µg/mL **1** in dilution buffer "A" (0.5% (w/v) BSA in TBS-T) followed by overnight incubation with rocking at 4 ºC. Wells were washed with TBS-T three times every 5 minutes. Then, 50 µL of primary antibodies (either commercial human serum (Sigma-Aldrich, H4522) or one of three clinical human serum samples from NYU Langone Center for Biospecimen Research and Development) were added at 1:10 dilution in dilution buffer A. Primary antibodies in each well were incubated at 25 º C for 1 hour with shaking. After discarding the primary antibodies, and washing the wells with 200 µL TBS-T three times every 5 minutes, 50 µL of secondary antibody (goat anti-human IgG (H+L) HRP, 1:50K, Invitrogen, 31410) in dilution buffer, was added into each well and incubated with shaking at 25 º C for 1 hour. Then, after discarding secondary antibodies and washing each well with 200 µL TBS-T six times every 5 minutes, residual TBS-T was removed and 50 µL of tetramethylbenzidine (TMB, ThermoFisher, 34028) was added to each well and incubated for 10 min at 25 ºC with shaking before quenching with 50 µL of 2 M sulfuric acid. Absorbance of wells was measured at 450 nm using a plate reader (SpectraMaxiD5, Molecular Devices) prior to analysis in SoftMax Pro 7.1.

For ELISA measurements of the binding interaction between **1** and IgG, IgA or IgM only, IgG mixture (Sigma-Aldrich I4506), IgA mixture (I4036) or IgM mixture (I8260) were used as the source of primary antibodies, whereas the noted anti-human IgG HRP antibody, goat anti-human IgA HRP (1:3000, Biolegend, 411002) or goat anti-human IgM (µ-chain specific) HRP (1:10K, Sigma Aldrich, A0420) were used as the secondary antibody, respectively, following the protocol above.

For ELISA measurements of the binding interaction between **1** and anti-*E. coli* LPS O16 or O25 antibody, rabbit anti-*E. coli* O16 or O25 LPS antibody were used as the source of primary antibody, respectively (titration of 1:50, 1:20 and 1:10 for each antibody in dilution buffer A, SSI Diagnostica, 85012 or 85022, respectively), whereas donkey anti-rabbit IgG (minimal x-reactivity) HRP (1:500, Biolegend, 406401) was used as secondary antibody for each. A similar protocol as described above was used for each.

For competition ELISA assays, each monosaccharide or LPS sample was stored in water and then serially diluted in TBS-T. Competitors were used at the following final concentrations: GlcNAc, Glc: 62.5, 125, 250, 500 mM; L-Rha plus or minus GlcNAc (combined concentration of sugars): 31.2, 62.5, 125, 250 and 500 mM; *E. coli* LPS O26, O25, O16 or Lipid A + core: 0.5, 1, 2, and 4 mg/mL). Competitors were added after the wash step following the addition of primary antibody (commercial human serum, 1:10 in buffer A), and added in 50 µL TBS-T total volume in each well followed by incubation at 25 ºC for 1 hour. The other steps were performed as described in the protocol above. A similar plate-based competition protocol utilizing LPS and immobilized glycoprotein was previously reported, except the plates were not coated with streptavidin.[9]

To evaluate an alternative LPS competition ELISA protocol, 4 mg/mL of LPS samples (*E. coli* LPS O25, O16 or Lipid A + core) were pre-incubated with primary commercial human serum antibodies (1:10 in buffer A) for 1 hr at 25 °C and incubated samples were added to plates after the wash step following the addition of **1** to each well. The other steps were performed as described in the protocol above. We found that this method led to non-specific competition. To evaluate if LPS binds to multiwell streptavidin-coated plates, 50 µL of 4 mg/mL of *E. coli* O16 LPS in dilution buffer A was added to the wells and incubated overnight with rocking at 4 °C after the initial wash step. Commercial human serum and goat anti-human IgM HRP were used as primary and secondary antibodies, respectively, following the protocol above.

**Human serum antibody enrichment using disaccharide probe 1.** 250 µL of slurry agarose streptavidin resin (Millipore Sigma) was loaded onto a frit column (Sigma Aldrich). $N_{2(g)}$ was flushed through the column to remove residual buffer (the column was flushed for all washing and elution steps described below). The column was further washed with 250 µL of PBS and this was repeated three times in total. 250 µL of 0.5 mg/mL **1** dissolved in PBS was loaded onto the column and was incubated for 1 hr at RT (25 º C) with rocking. 250 µL of 0.05 % Tween-20 in PBS (PBST20) was then added to the column to reduce non-specific binding to the resin, followed by two washes with 250 µL PBS. 2.5 mL of commercial human serum (Sigma-Aldrich H4522) was loaded onto the column and incubated at 4°C overnight with rocking. The flowthrough was then discarded, and the column was washed with PBST20 followed by three consecutive washes with PBS to remove unbound serum components (250 µL was used for each wash). To elute the enriched antibody solution, 250 µL of 500 mM L-Rha in PBS was applied to the column and incubated for 1 hr at 4°C. The flowthrough was collected, and the crude antibody absorbance was measured at 280 nm (NanoDrop One, Thermo Scientific). Note that in some experiments, additional eluate fractions were collected using 2 x 250 µL of 500 mM L-Rha, followed by 3 x 250 uL of 500 mM GlcNAc in PBS, and the crude absorbance of each was measured at 280 nm. The first eluate using each sugar was used for relevant experiments. The enriched serum samples were aliquoted and stored in the elution buffer supplemented with 0.1% $NaN_3$ at -80 °C. Aliquots were thawed prior to use for dot blots.

**Dot blots of bacterial strains with RGB-enriched human serum.** 5 mL of cell cultures: *M. smegmatis* (Msm), *E. coli wbbL*- (Lipid A + core, O-Ag-), O16, O25, O55, and O157 (see **Table S3**) were grown to mid-log phase from overnight cultures, and the cells were normalized to an OD600 = 5 in PBS (note that OD580 measurements were used for *M. smegmatis*). 1:1 to 1:32 cell dilutions were prepared in PBS by two-fold serial dilutions and 2 µL of cells were spotted on a nitrocellulose membrane (Bio-Rad) and were left to dry completely. The membranes were equilibrated in blocking buffer (2.5 % (w/v) non-fat dry/skim milk powder (bioPLUS Chemicals) in TBS-T) for 1 hr at room temperature (25 º C). Membranes were then incubated overnight at 4 ºC in 1:1000 anti-**1** "enriched" serum in blocking buffer (typically, L-Rha-eluted samples were used, unless it was noted that GlcNAc-eluted samples were used instead). Control blots were instead carried out with 1:1000 anti-O25 antibody or 1:2000 anti-O16 antibody (SSI Diagnostica) at this step. The membranes were then washed with TBS-T (t = 3 hr) with rocking, and, when indicated,

washed with TBS-T containing 500 mM GlcNAc (t = 3 hr) with rocking. Secondary antibody (Goat anti-Human IgG HRP, 1:50K, Invitrogen) in 2.5 % blocking buffer was then added at room temperature (25 ºC) for 1 hr. For serotyping controls (anti-O25 and -O16) donkey anti-rabbit IgG HRP, 1:3K, BioLegend) was used instead. The membranes were finally washed for 3 hr in TBS-T prior to imaging with Clarity Max Western ECL Substrate (Bio Rad).

To evaluate the specificity of **RGB**-enriched serum binding to bacterial strains, dot blot experiments were carried out as described in the paragraph above except the following difference was introduced: 500 mM of either L-Rha or biotin or no competitor was pre-incubated with 1:1000 anti-**1** "enriched" serum in blocking buffer (25 °C, t = 1 hr) before addition to membranes overnight at 4 °C. The remaining steps were carried out as described.

**Table S3. List of bacterial strains used for LPS extraction and dot blot experiments.**

| No. | Strain Name | Strain information | Source/Reference |
|---|---|---|---|
| 1 | *E. coli* MG1655 *wbbL*- (*wbbL::*IS5) | Wildtype *E. coli* strain containing a genetic insertion in *wbbL* (provides Lipid A + core or O-Ag (-)) | Gift from Rojas Lab (NYU) |
| 2 | *E. coli* MG1655 *wbbL*+ | *E. coli wbbL*+ O16-producing strain | Gift from the Jorgenson lab[8] (UAMS) |
| 3 | *E. coli* O18 ab:K-:H14 | *E. coli* O18ab:K-:H14 producing strain (NR-17676) | BEI Resources |
| 4 | *E. coli* O25:H4 | *E. coli* O25:H4 producing strain (NR-17661) | BEI Resources |
| 5 | *E. coli* O55:H7 | *E. coli E. coli* O55:H7producing strain (NR-20450) | BEI Resources |
| 6 | *E. coli* O157:H7 | *E. coli* O157:H7 (NR-6) | BEI Resources |
| 7 | *M. smegmatis* mc$^2$155 | *M. smegmatis* (Msm) wild-type strain | Gift from the Glickman lab (Memorial Sloan Kettering Institute) |

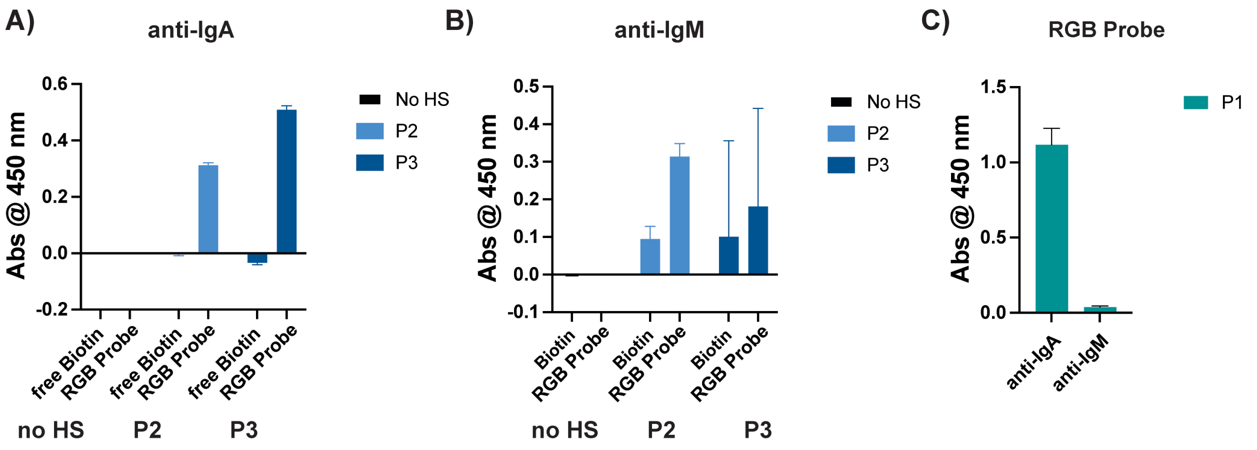


**Figure S1. ELISA analysis of human antibody isotypes using clinical samples show different levels of IgA and IgM.** ELISA analysis using (A) anti-human IgA and (B) anti-human IgM antibodies against de-identified clinical human serum samples (P2 and P3) with biotin control versus the **RGB** probe. (C) ELISA analysis of clinical human serum sample P1 using anti-human IgA and anti-human IgM antibodies (additional controls were not performed due to limited P1 serum sample). Each dataset has a "no probe" replicate subtracted to account for primary/secondary antibody binding to the plate, n = 3, error bars represent standard error of the mean (SEM). "No HS" represents a "no human serum" control for parts A and B. As shown in **Figure 2B**, anti-IgG typically gave the highest signal across samples.

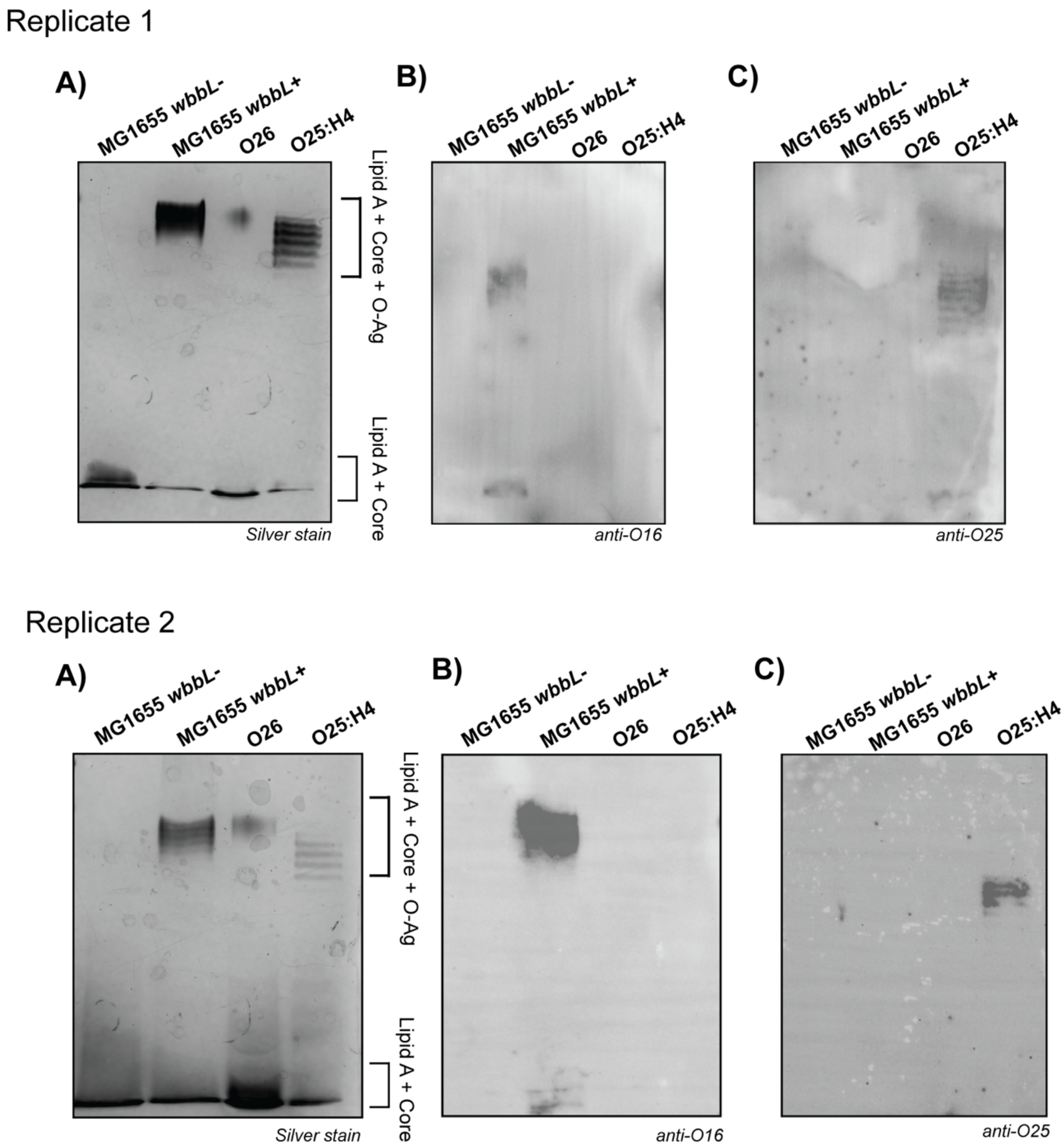


**Figure S2. Biological replicates of silver stain, O-16 and O-25 blots following analysis of LPS extracted from *E. coli* strains and commercial LPS.** LPS from *E. coli* MG1655 *wbbL*- (Lipid A + core oligosaccharides or O-Ag(-)), *E. coli* MG1655 *wbbL*+ (O16), and *E. coli* O25:H4 were extracted as described in Methods. 15 µL of extracted LPS and 10 µg of commercially available *E. coli* O26 LPS (from *E. coli* O26:B6, Sigma**,** 93572-42-0) were analyzed by SDS-PAGE and (A) visualized by silver stain, (B) anti-O16 immunoblotting, and (C) anti-O25 immunoblotting.

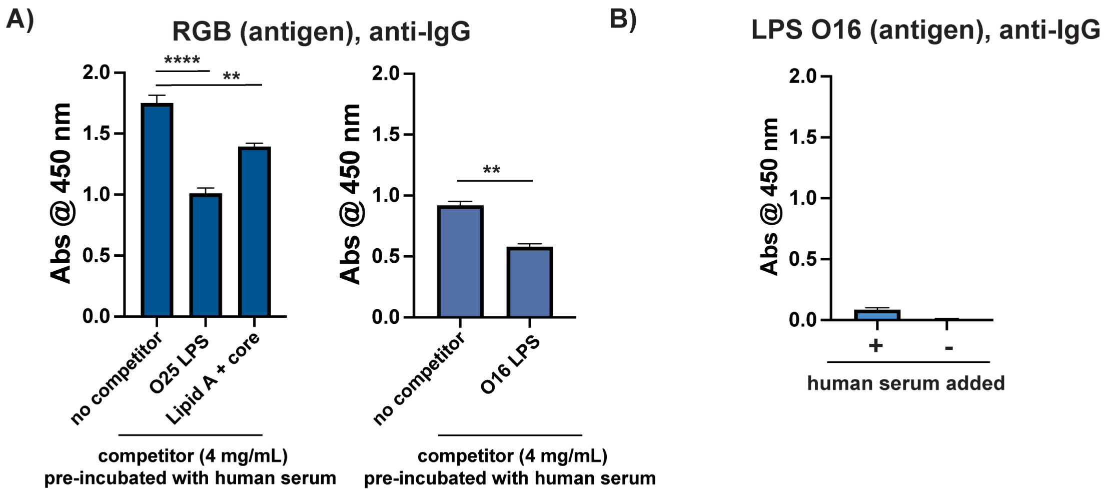


**Figure S3. LPS does not show sequence-dependent competition with antibodies when pre-incubated prior to addition to RGB.** (A) Competition ELISA experiments in which indicated purified LPSs were pre-incubated with commercial human serum (t = 1 hr, 25 ºC) prior to addition to **RGB**-bound to streptavidin-coated plates. Decreased IgG binding is observed with all LPSs tested (even LPS O-Ag(-), called “Lipid A + core”). These results suggest that the pre-incubation protocol leads to non-specific competition, perhaps due to the long lipid chains present in LPS. Compare to optimized experiments shown in **Figure 2C-D,** in which competitors were added to plates after human serum. (B) ELISA analysis using LPS O16 (4 mg/mL) as an antigen with or without commercial human serum added as a primary antibody followed by detection using anti-human IgG antibodies indicates that LPS alone does not bind added antibodies. Each dataset has a “no antigen” replicate subtracted to account for primary/secondary antibody binding to the plate (n = 3, error bars represent SEM, $^{**}p<0.01$; $^{****}p<0.0001$, paired t-test used).

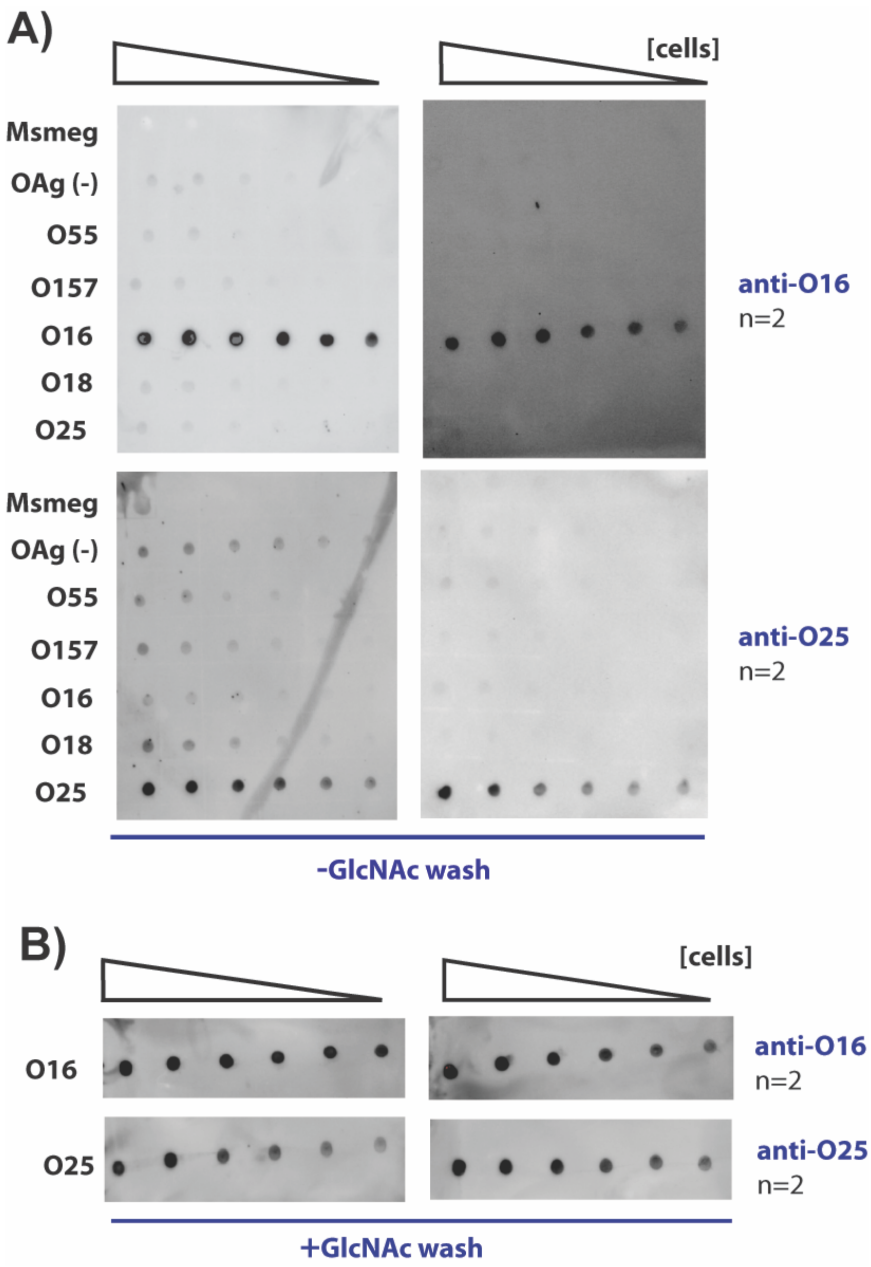


**Figure S4. Additional replicates of dot blot experiments using commercial serotyping antibodies.** Blots using commercial anti-O16 and anti-O-25 as the primary antibody source against indicated bacterial strains in the (A) absence of a GlcNAc wash step versus (B) the presence of GlcNAc wash step during the dot blot protocol (see **Figure 3B**). Note that one of each of the blots in part A was truncated to give the anti-O16 and anti-O25 blot shown in **Figure 3C**(*ii*) (n = 2 overall for each condition). Msmeg or Msm is *M. smegmatis*.

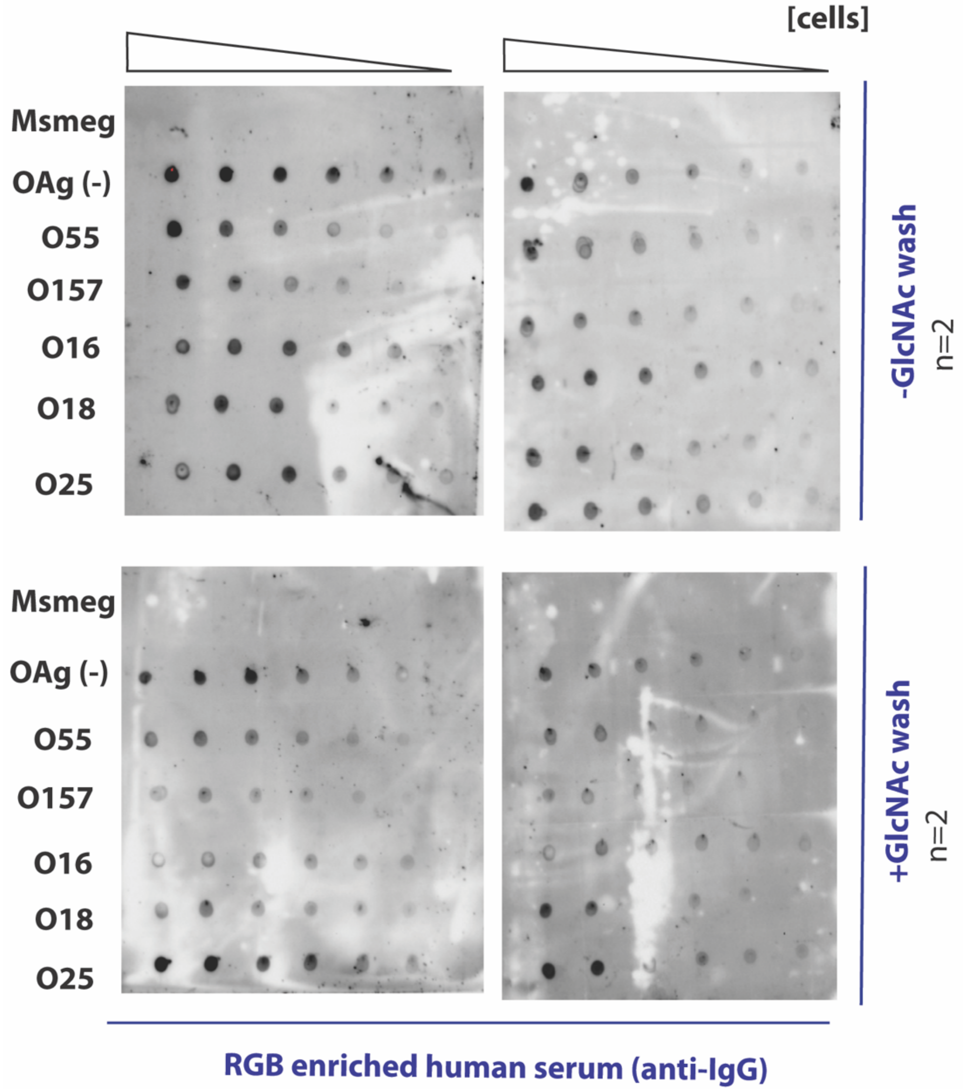


**Figure S5. Additional replicates of dot blot experiments using enriched human serum -/+ GlcNAc wash.** Blots using **RGB**-enriched human serum as the primary antibody source against indicated bacterial strains in the absence of a GlcNAc wash step (top panel) and presence of a GlcNAc wash step (bottom panel) of the dot blot protocol (see **Figure 3B**). These replicates were performed in addition to the experiments shown in **Figure 3C(*i*)** (n = 3 overall for each condition).

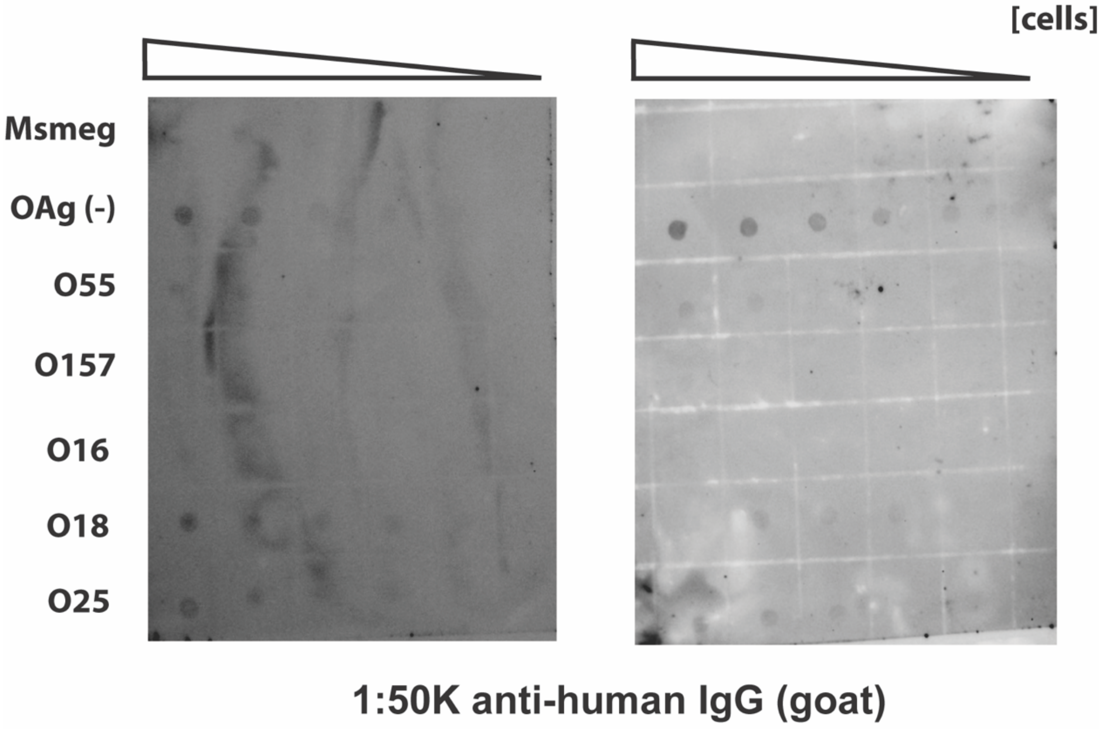


**Figure S6. Control dot blot analyses using only secondary antibody.** Replicate dot blots were performed as described for indicated strains using only the secondary antibody (anti-human IgG HRP) with no human serum added. Note that there is some non-specific detection of bacteria, especially *E. coli* O-Ag(-), which contains only Lipid A + core oligosaccharides (n = 2).

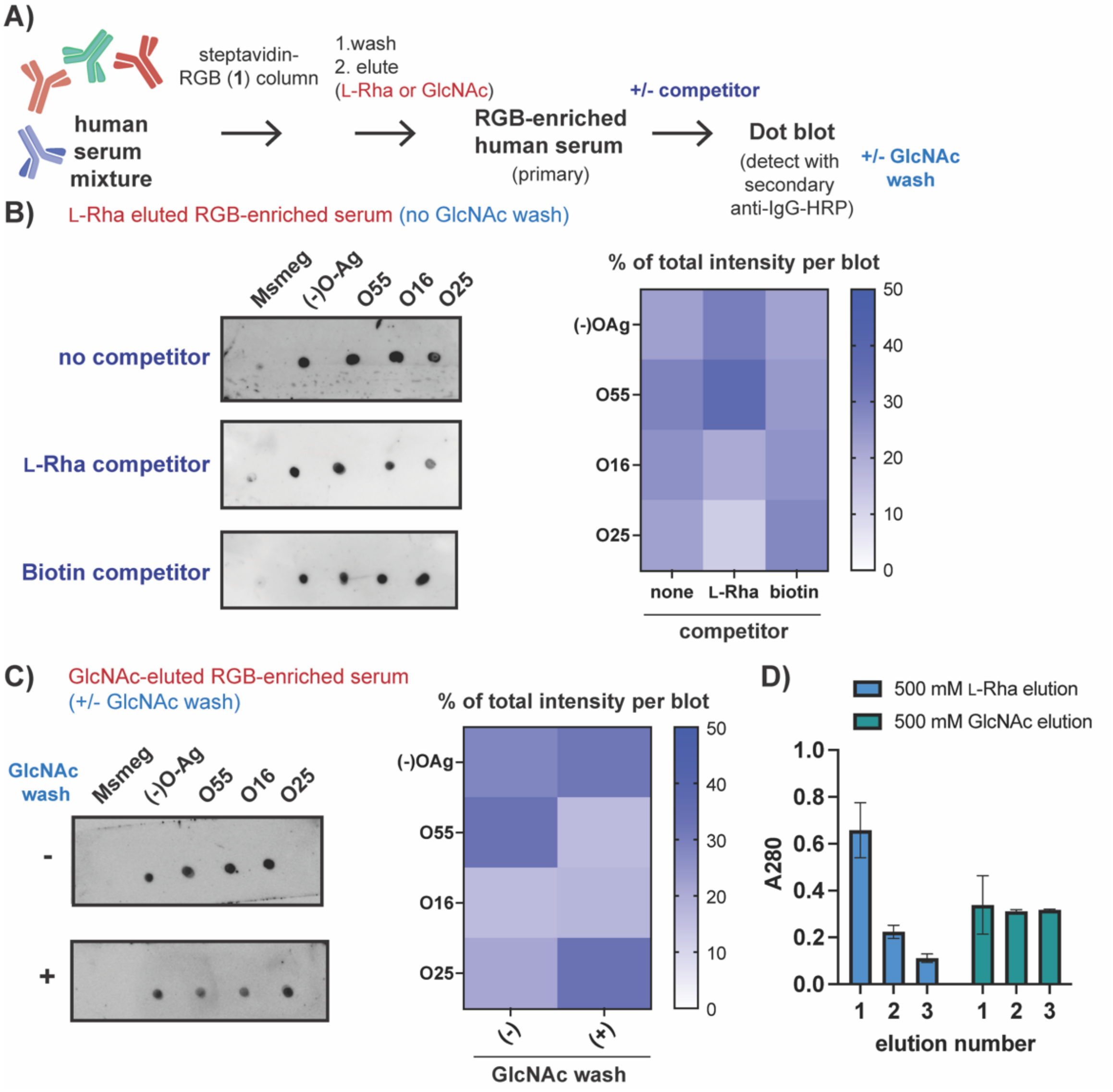


**Figure S7. Additional dot blot analyses suggest that detection of *E. coli* by RGB-enriched serum is L-Rha-dependent.** (A) Schematic of additional protocol to enrich commercial human serum antibodies with **RGB**; steps that were varied are indicated in different colors. (B) Representative dot blot analysis (left) and corresponding quantification (right) of indicated bacterial strains using **RGB**-enriched serum following elution with excess L-Rha followed by pre-incubation with different competitors. Compared to no competitor or excess biotin (500 mM), preincubation (1 hr, 25 ºC) of L-Rha (500 mM) with enriched human serum primary antibody mixture leads to a decrease in signal detection for *E. coli* O25 (~12% of the total signal intensity compared to ~22% for no competitor added). Biotin addition does not lead to dramatic changes in bacterial detection suggesting that bound antibodies do not interact with the biotin component of **RGB**; together, these data suggest *E. coli* O25 detection is L-Rha-dependent. (C) Representative dot blot analysis (left) and corresponding quantification (right) using **RGB**-enriched serum following elution with excess GlcNAc without and with an added GlcNAc wash step. Antibodies eluted with GlcNAc appear to bind various bacterial strains with and

without O-Ag, even when a GlcNAc wash step is used. (D) Absorbance at 280 nm (A280) measurements of eluate from immobilized **RGB** incubated with commercial human serum and eluted with 3 x 250 µL 500 mM L-Rha followed by 3 x 250 µL 500 mM GlcNAc indicates antibodies are eluted by both sugars (n = 2, error bars represent standard deviation). The first eluate with each sugar was used for experiments shown in parts B and C, respectively. Note for parts B and C, only undiluted cell cultures were used for dot bot analysis.

**References:**

(1) Frihed, T. G.; Pedersen, C. M.; Bols, M. Synthesis of All Eight Stereoisomeric 6-Deoxy-L-Hexopyranosyl Donors - Trends in Using Stereoselective Reductions or Mitsunobu Epimerizations. *European J. Org. Chem.* **2014**, *2014* (35), 7924–7939.

(2) Chaudhury, A.; Maity, S. K.; Ghosh, R. Efficient Routes toward the Synthesis of the D-Rhamno-Trisaccharide Related to the A-Band Polysaccharide of Pseudomonas Aeruginosa. *Beilstein J. Org. Chem.* **2014**, *10*, 1488–1494.

(3) Wu, X.; Gao, L.; Sun, J.; Hu, X. Y.; Wang, L. Stable Pillar[5]Arene-Based Pseudo[1]Rotaxanes Formed in Polar Solution. *Chinese Chem. Lett.* **2016**, *27* (11), 1655–1660.

(4) Ludwig, A. K.; Michalak, M.; Xiao, Q.; Gilles, U.; Medrano, F. J.; Ma, H.; FitzGerald, F. G.; Hasley, W. D.; Melendez-Davila, A.; Liu, M.; Rahimi, K.; Kostina, N. Y.; Rodriguez-Emmenegger, C.; Möller, M.; Lindner, I.; Kaltner, H.; Cudic, M.; Reusch, D.; Kopitz, J.; Romero, A.; Oscarson, S.; Klein, M. L.; Gabius, H. J.; Percec, V. Design–Functionality Relationships for Adhesion/ Growth-Regulatory Galectins. *Proc. Natl. Acad. Sci. U. S. A.* **2019**, *116* (8), 2837–2842.

(5) Basu, N.; Mukherjee, M. M.; Ghosh, R. Synthetic Routes toward the Trisaccharide Related to the Lipopolysaccharide of Burkholderia Sp. HKI-402 (B4). *RSC Adv.* **2014**, *4* (96), 54084–54090.

(6) Davis, M. R.; Goldberg, J. B. Purification and visualization of lipopolysaccharide from Gram-negative bacteria by hot aqueous-phenol extraction. *J. Vis. Exp.* **2012**, (63), 3916.

(7) C. M. Tsai and C. E. Frasch, “A sensitive silver stain for detecting lipopolysaccharides in polyacrylamide gels,” *Anal. Biochem*. **1982**, *119*, 115–119.

(8) Jorgenson, M. A.; Young, K. D. Interrupting Biosynthesis of O Antigen or the Lipopolysaccharide Core Produces Morphological Defects in Escherichia coli by Sequestering Undecaprenyl Phosphate. *J. Bacteriol.* **2016**, *198* (22), 3070-3079.

(9) Kim, H.; Lupoli, T. J. Defined Glycan Ligands for Detecting Rare L-Sugar-Binding Proteins. *J Am Chem Soc* **2025**, *147* (14), 11693-11699.

$^1$H-NMR (400 MHz, $CDCl_3$) Compound **2**:

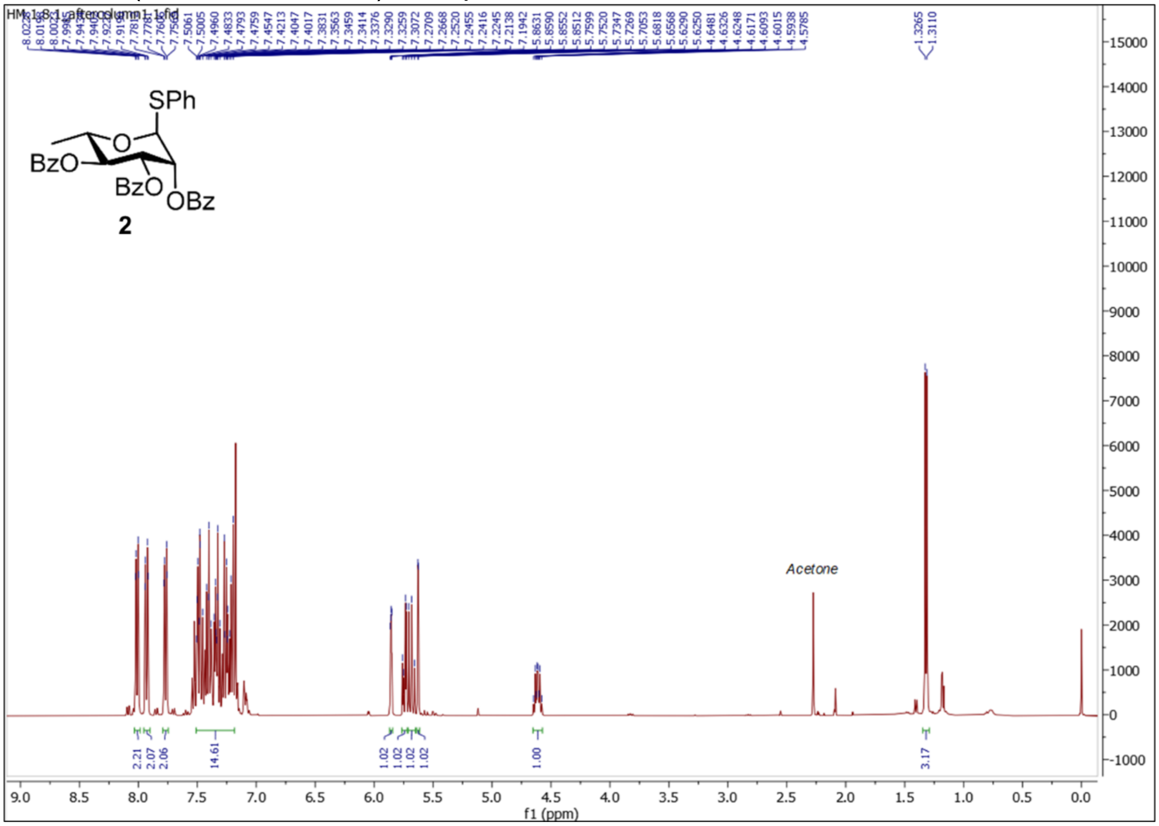

$^{13}$C{1H} NMR (101 MHz, $CDCl_3$) Compound **2**:

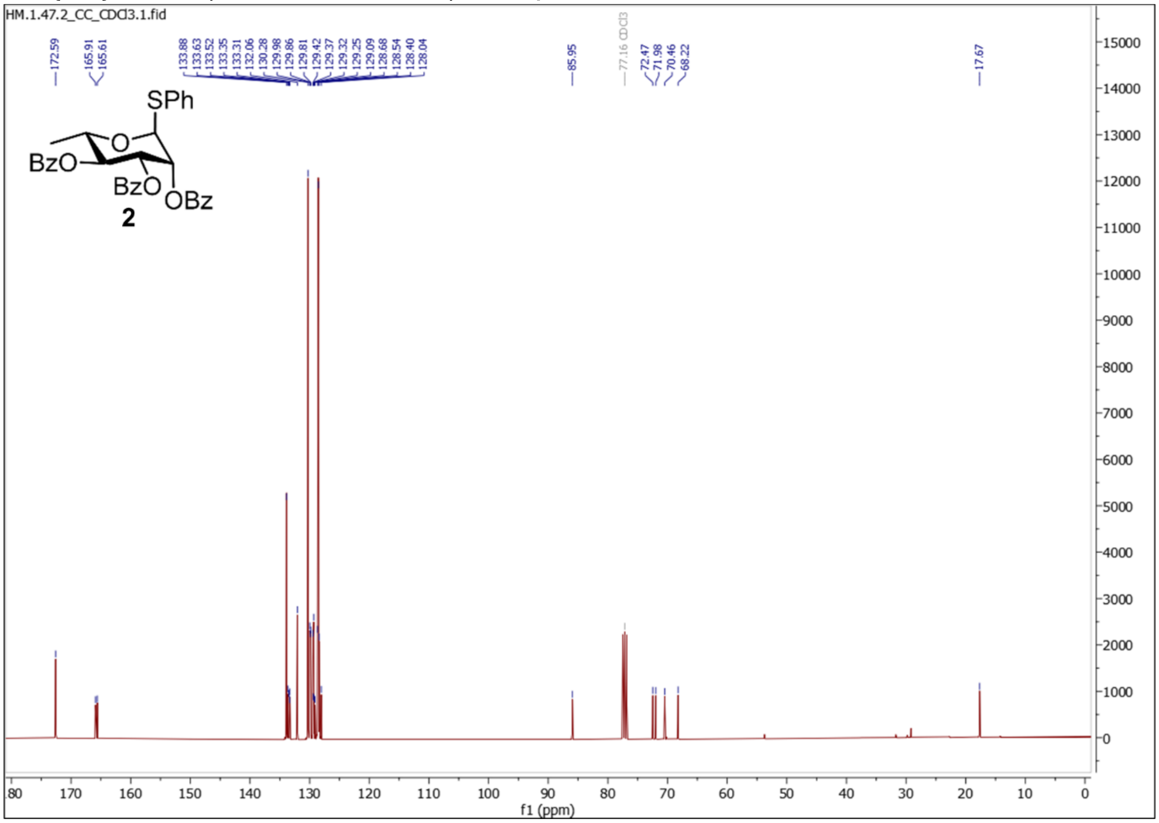

$^{1}H$-NMR (400 MHz, $CDCl_3$) Compound **3**:

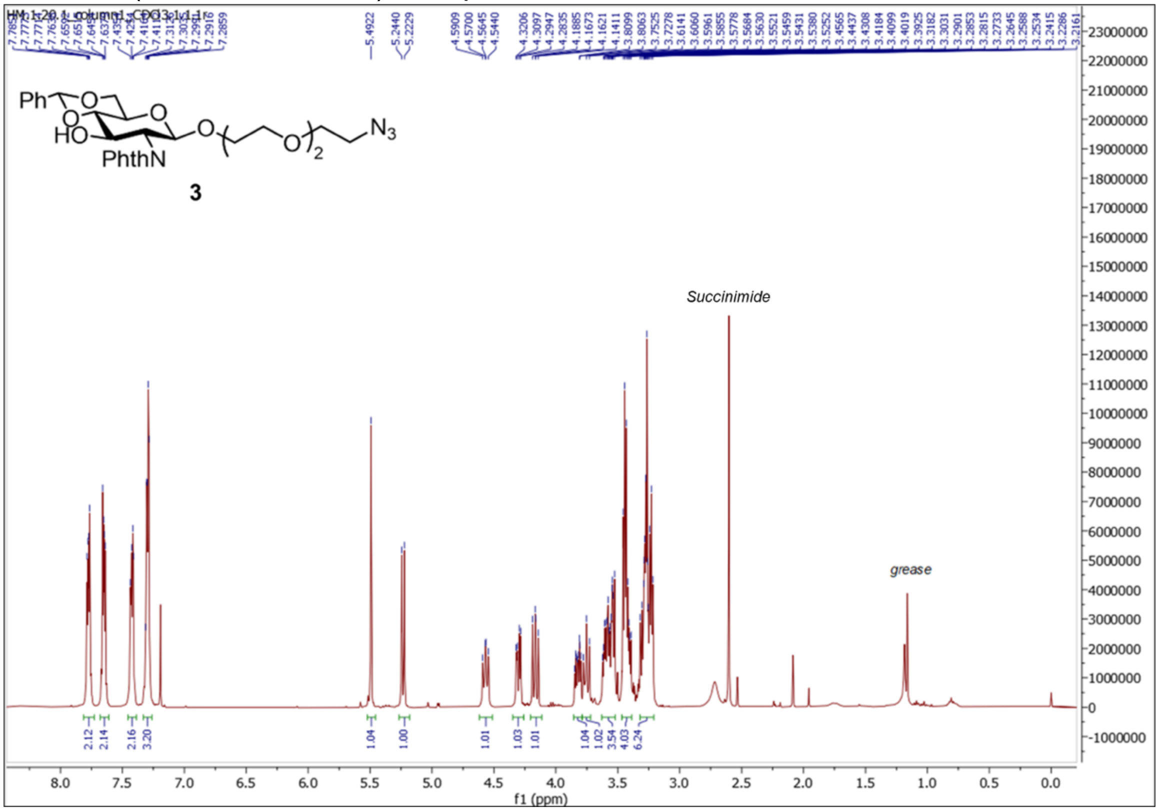


$^{13}C\{1H\}$ NMR (101 MHz, $CDCl_3$) Compound **3**:

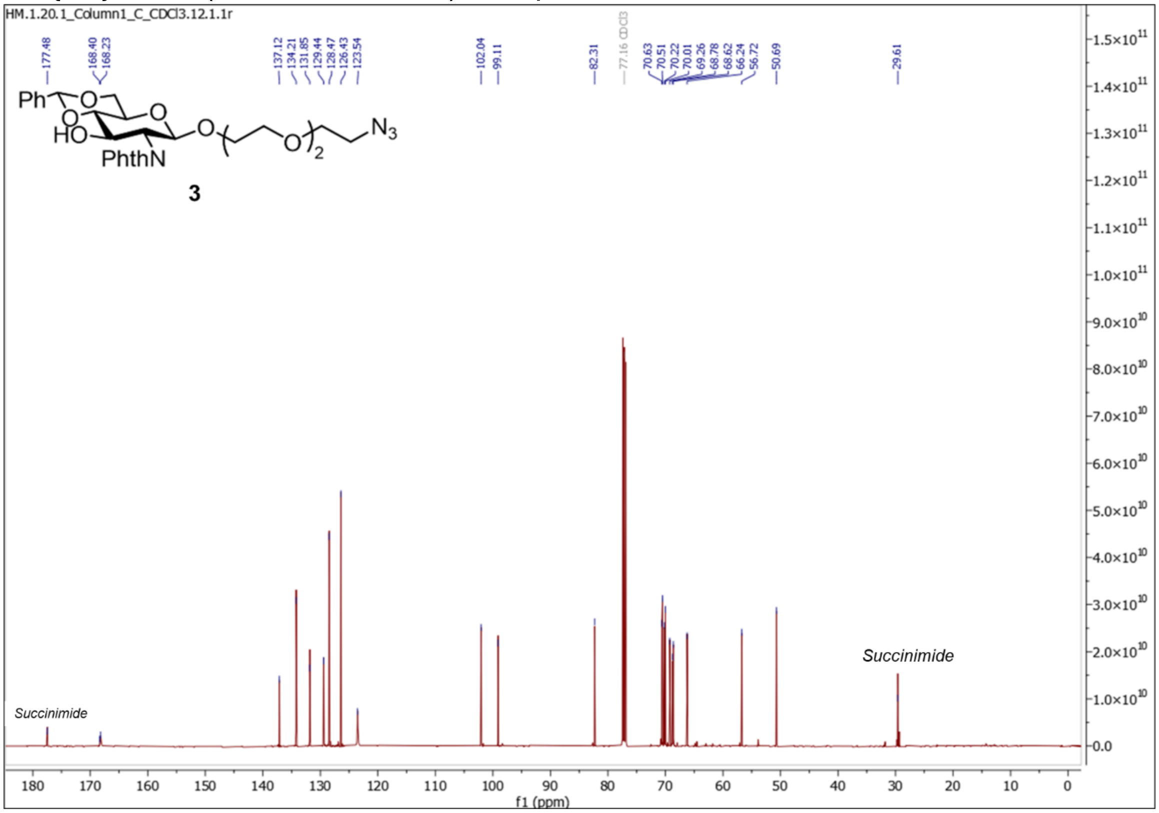

$^{1}$H-NMR (400 MHz, $CDCl_3$) Compound **6**:

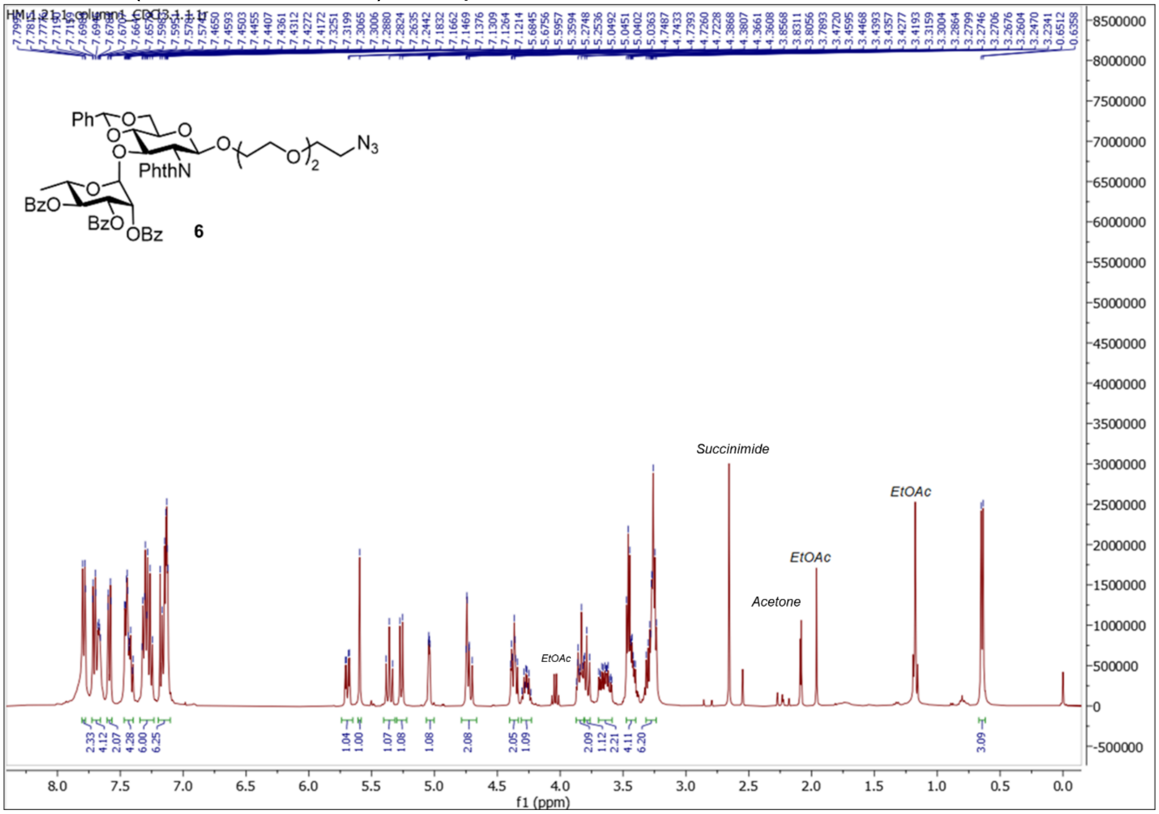


$^{13}$C{1H} NMR (101 MHz, $CDCl_3$) Compound **6**:

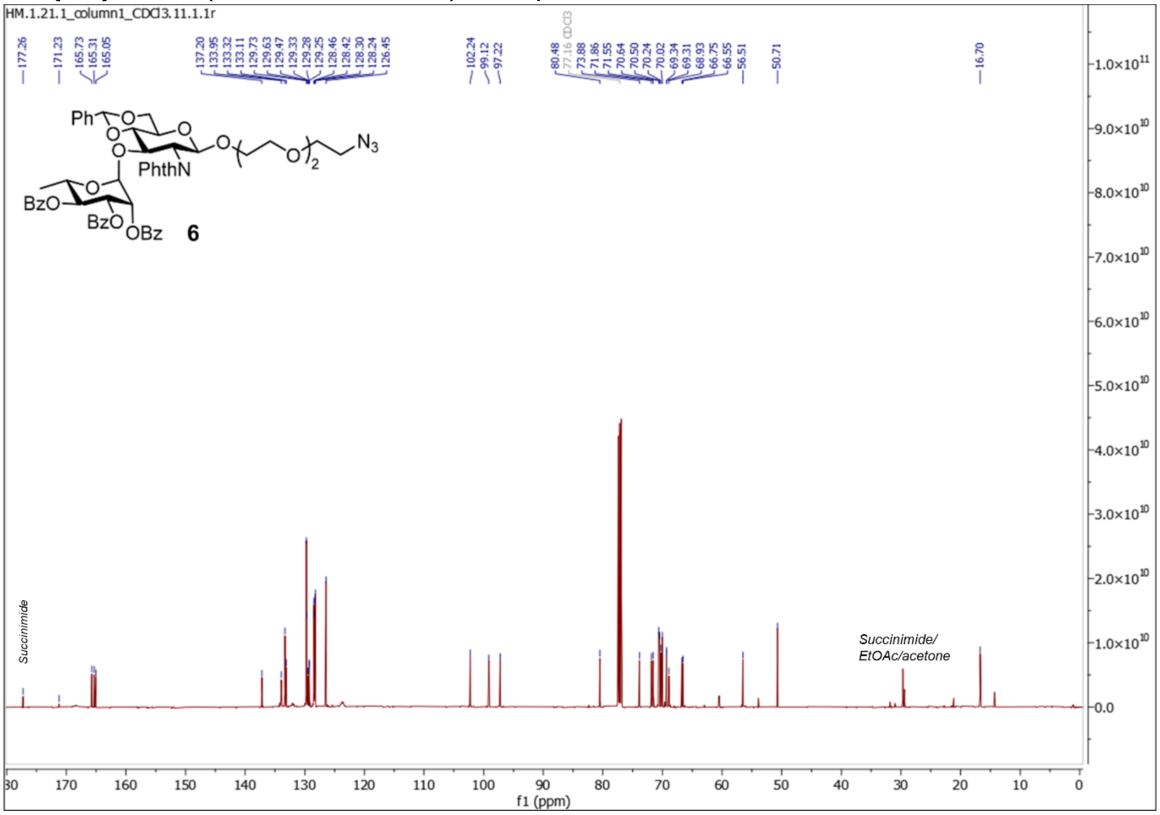

$^1$H-$^{13}$C HSQC compound **6**:

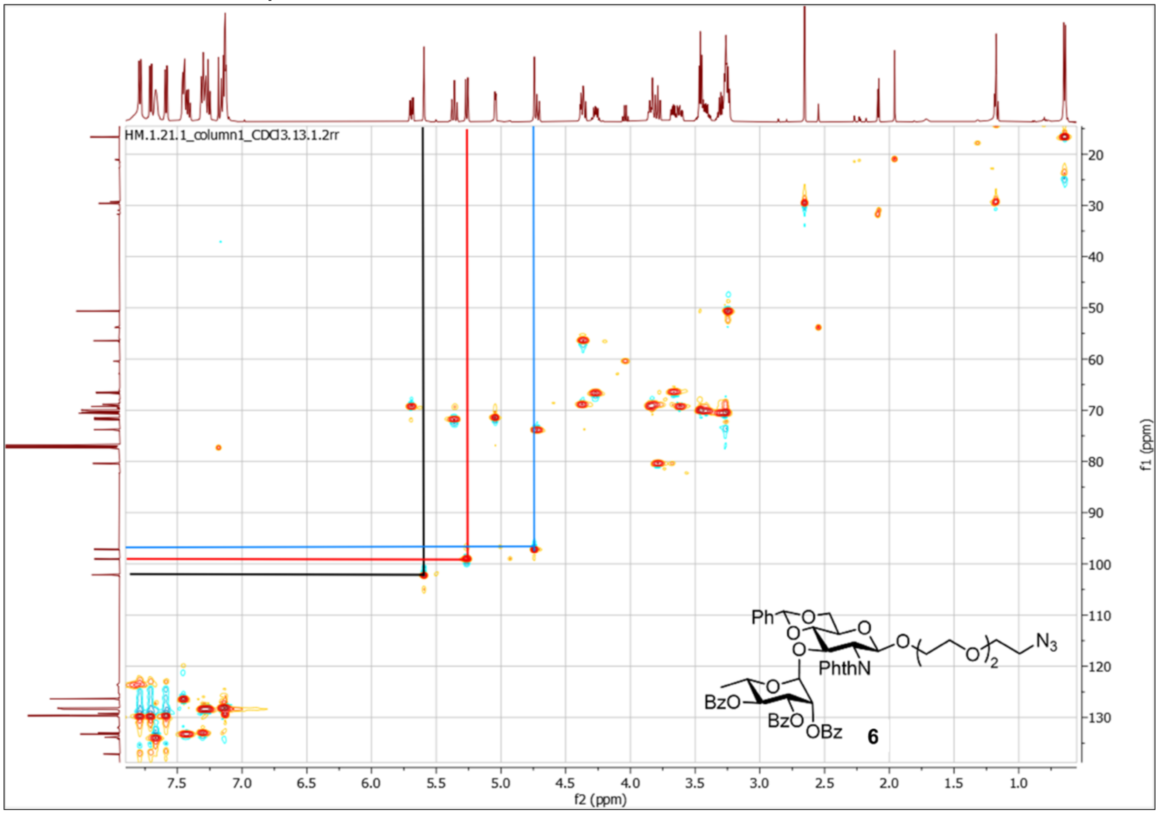

$^{1}$H-NMR (400 MHz, $CDCl_3$) Compound **7**:

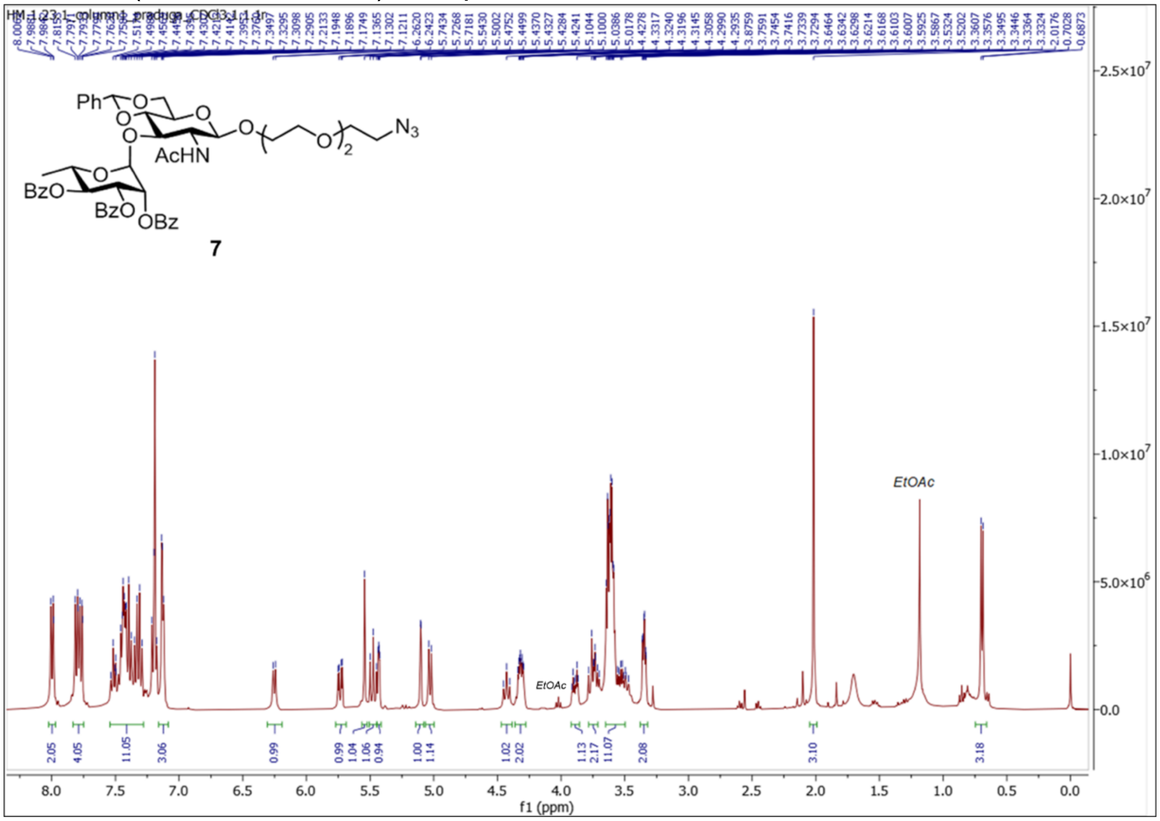

$^{13}$C{1H} NMR (101 MHz, $CDCl_3$) Compound **7**:

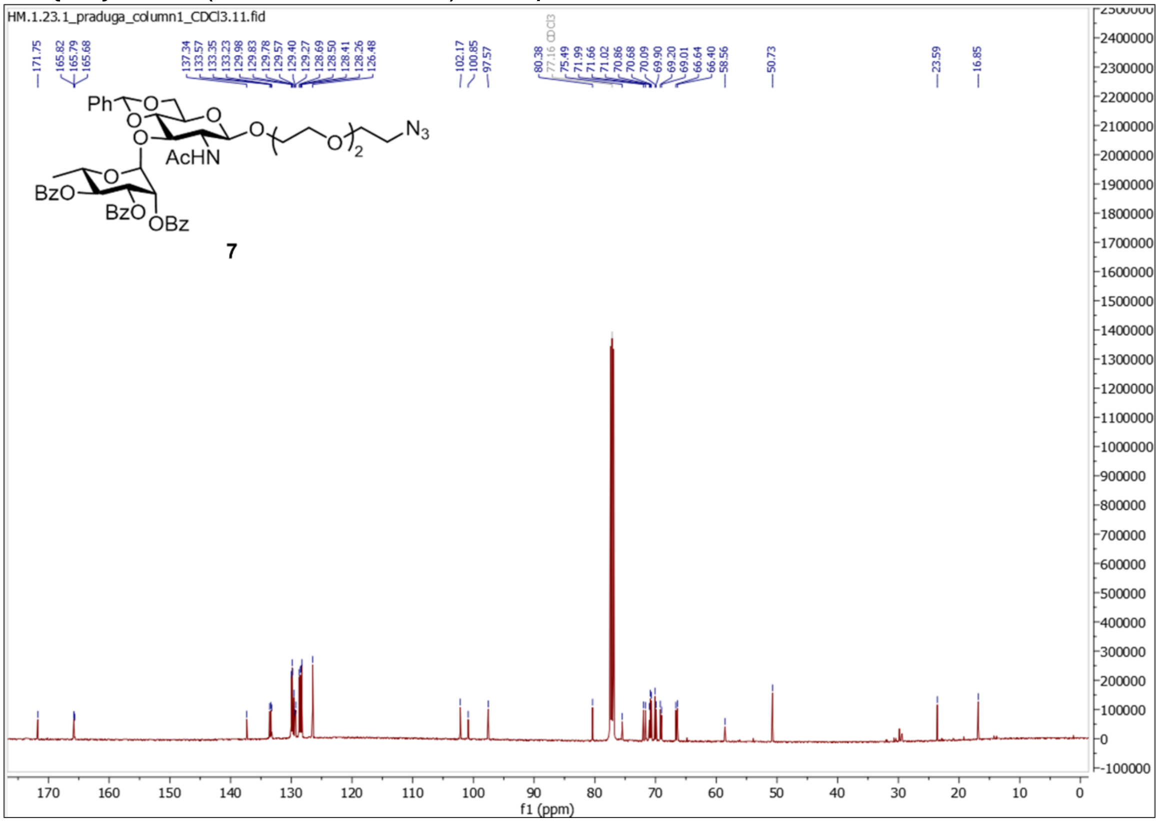

$^1H$-$^{13}C$ HSQC compound **7**:

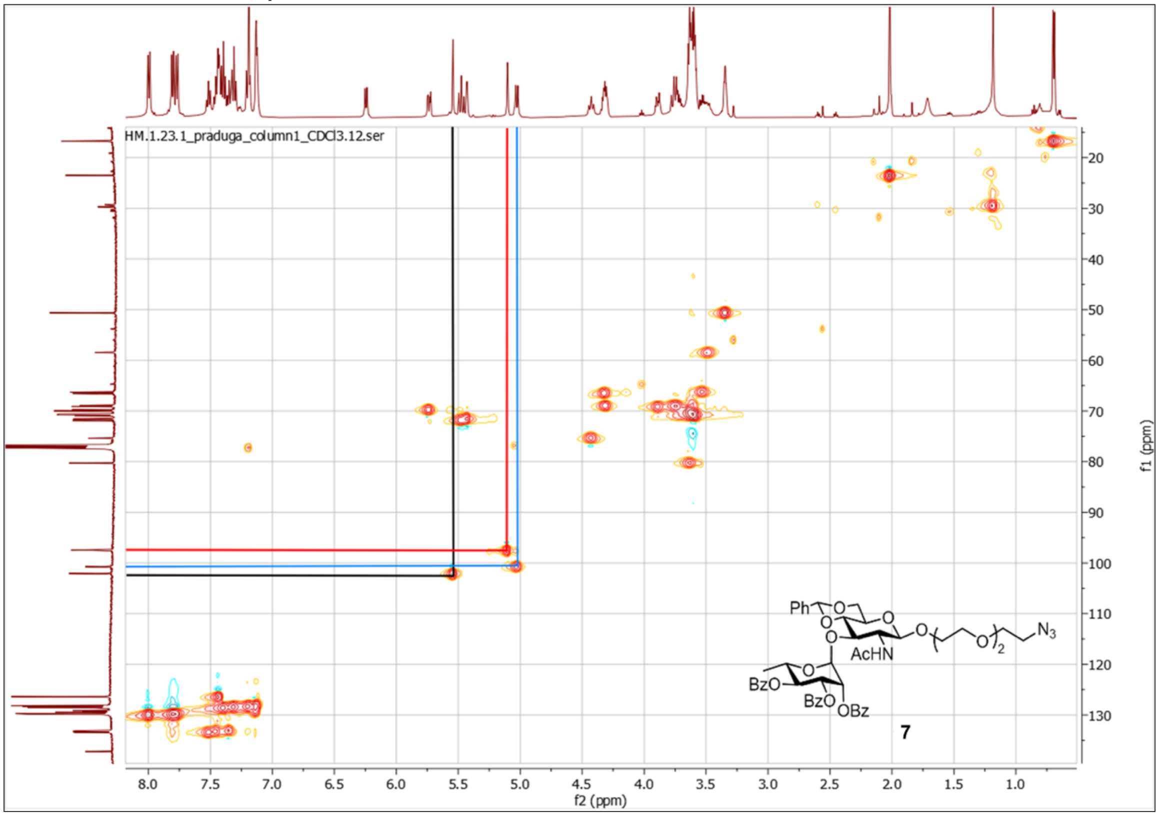

$^{1}$H-NMR (500 MHz, $CD_3OD$) Compound **8**:

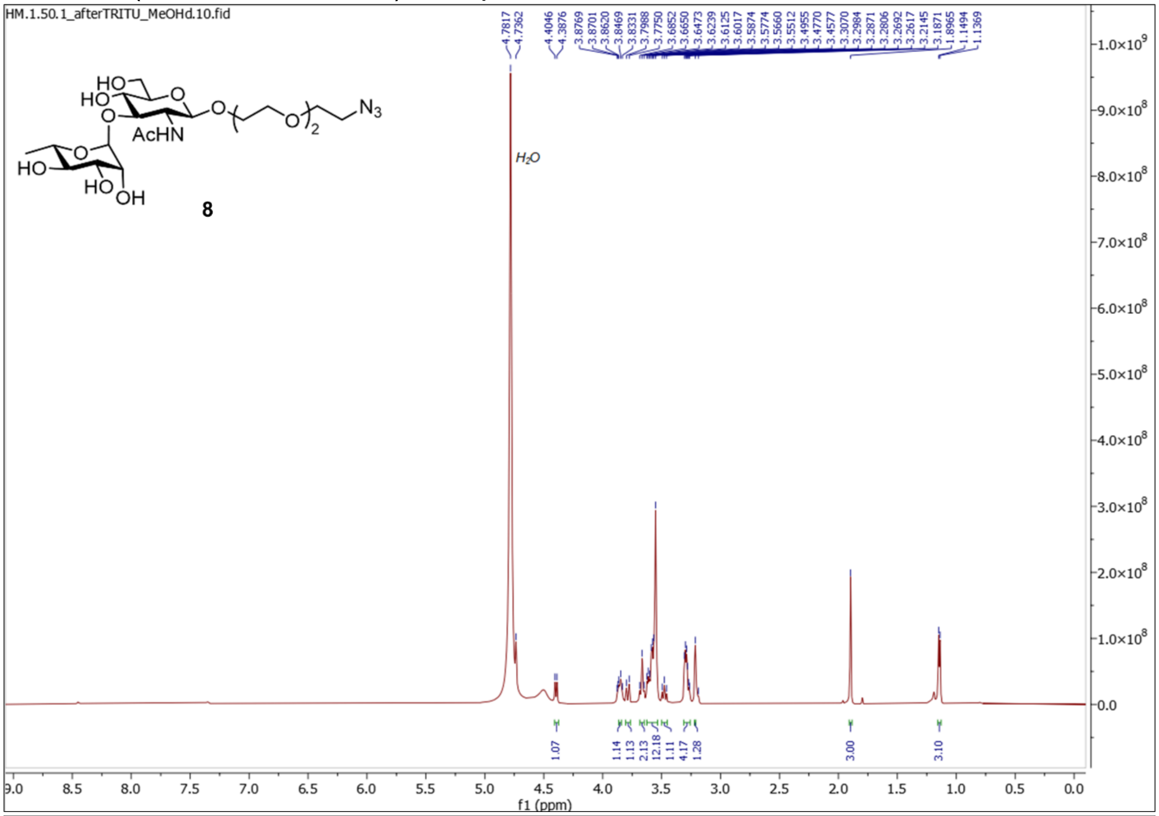


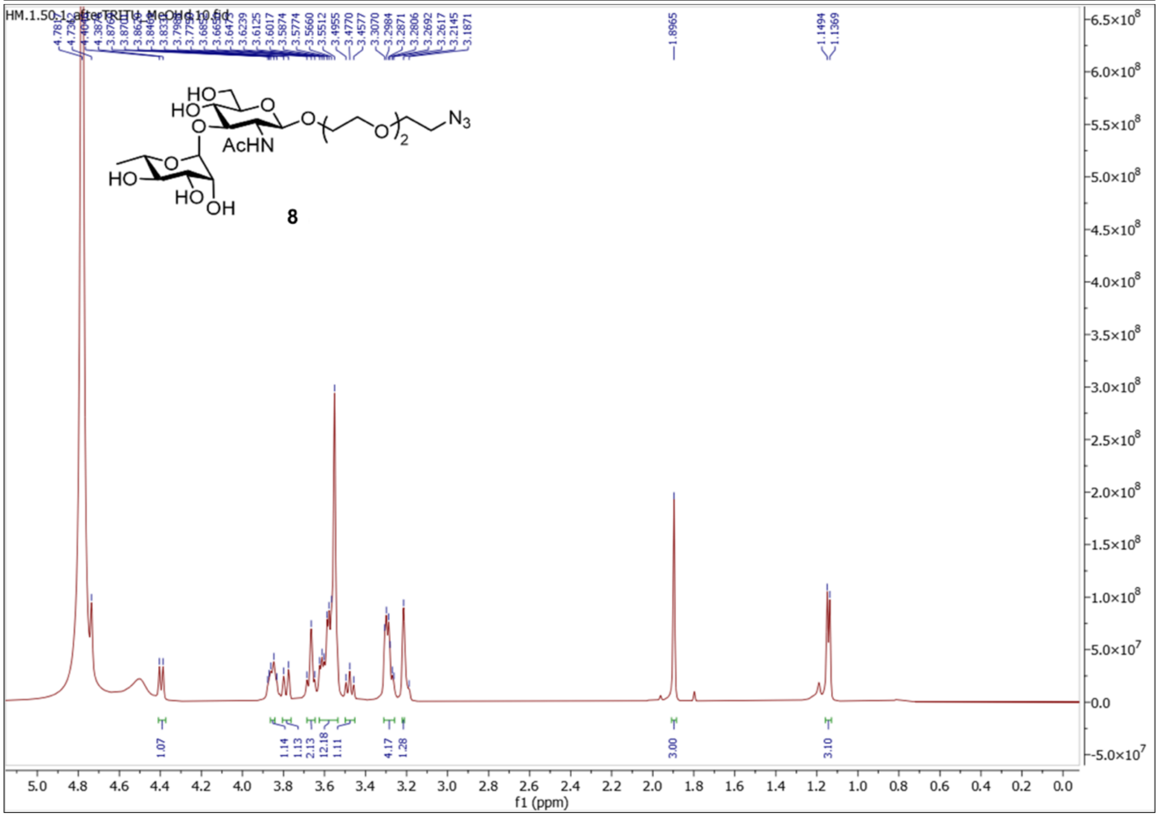

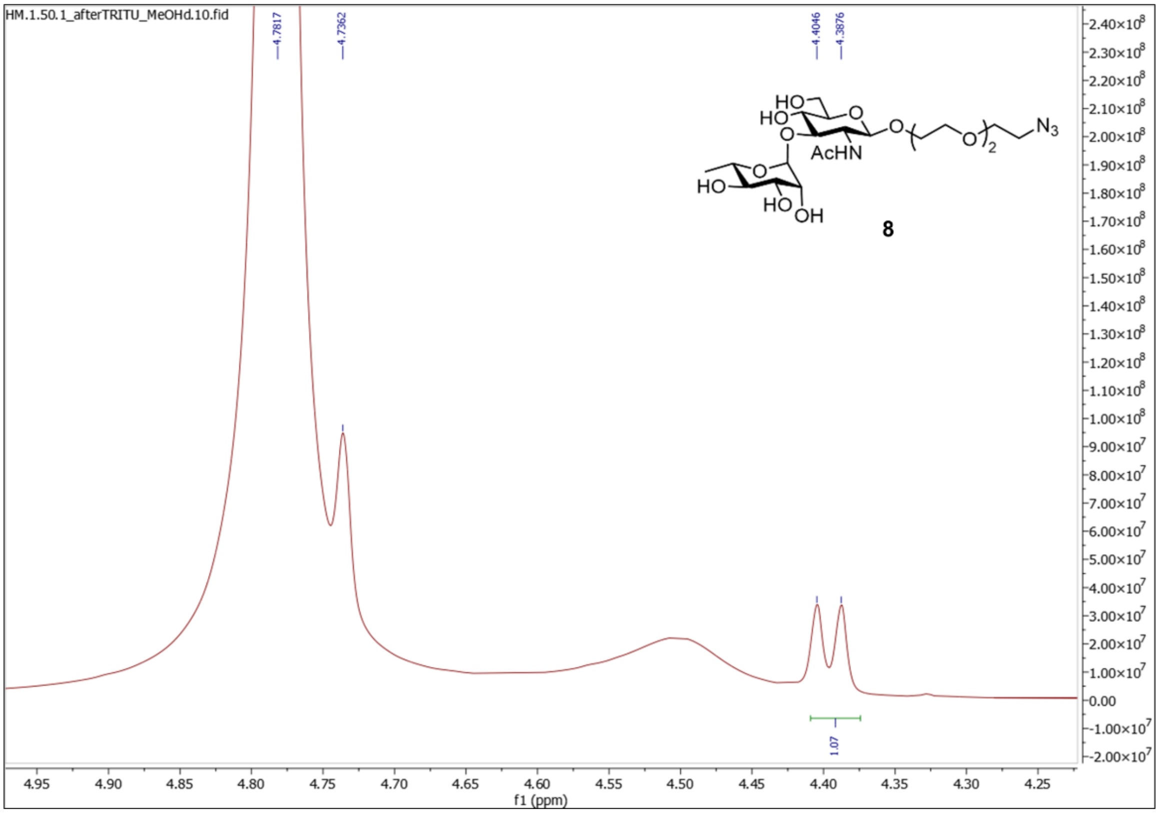


$^{13}C\{^1H\}$ NMR (125 MHz, $CDCl_3$) Compound **8**:

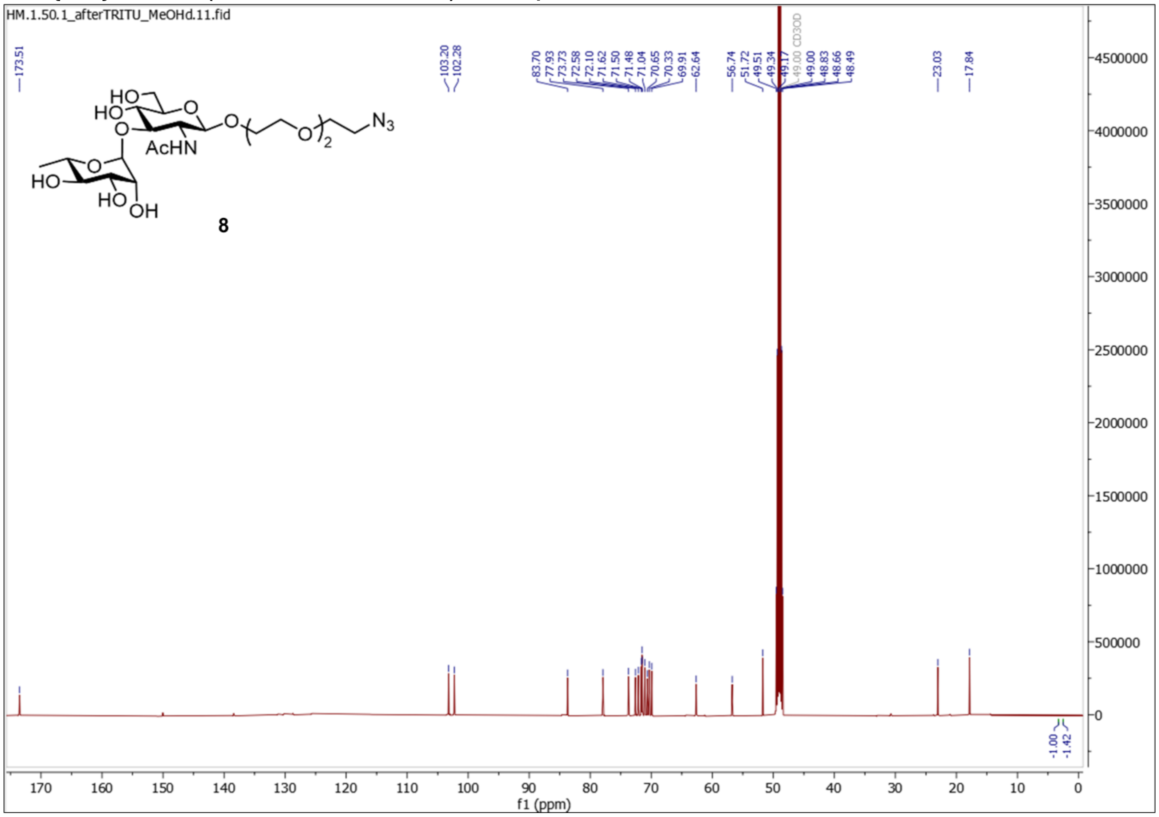

DEPT 135° compound **8**:

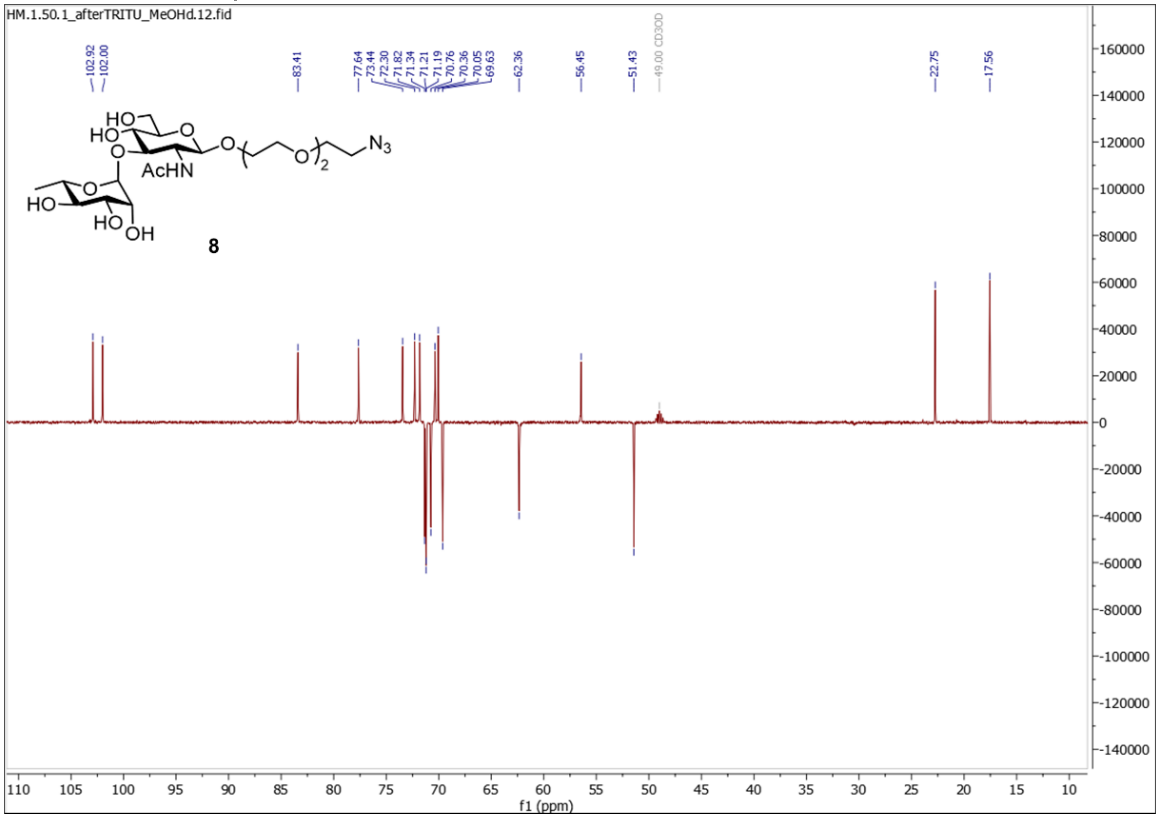


$^{1}H$-$^{13}C$ HSQC compound **8**:

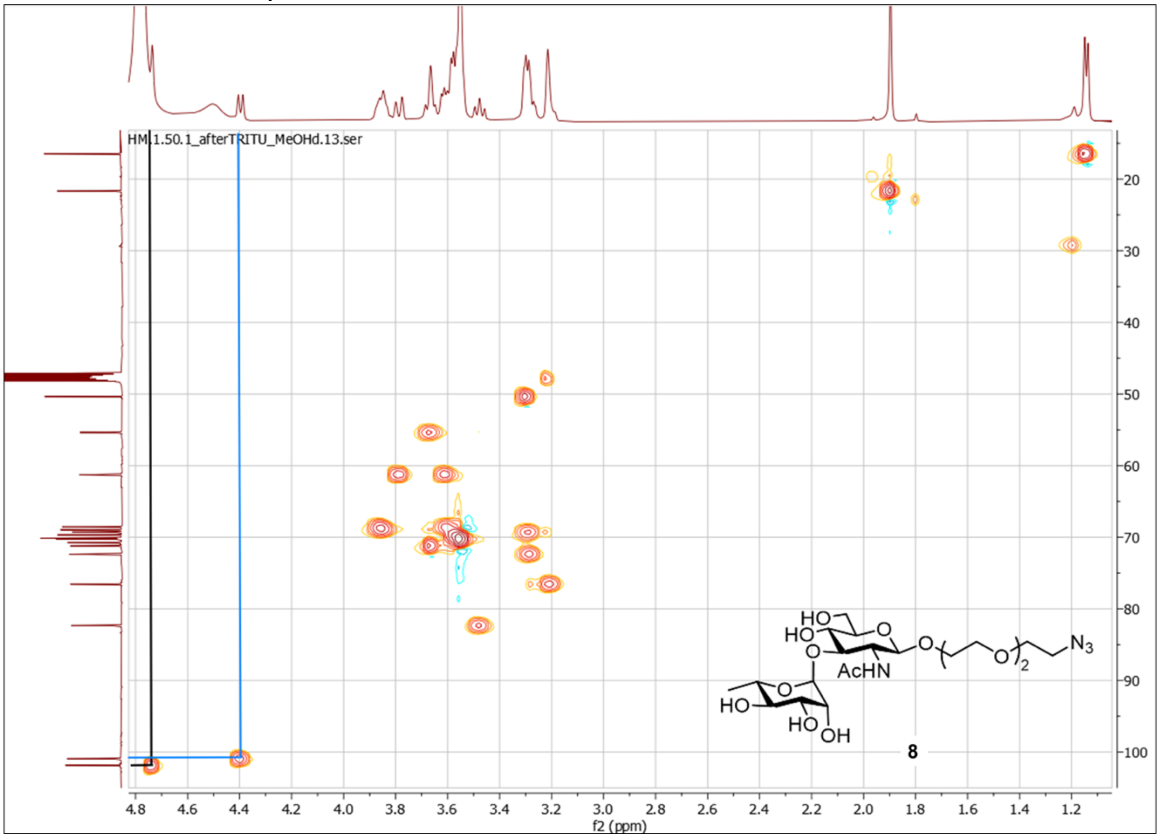

$^1$H-NMR (500 MHz, $CD_3OD$) Compound **1**:

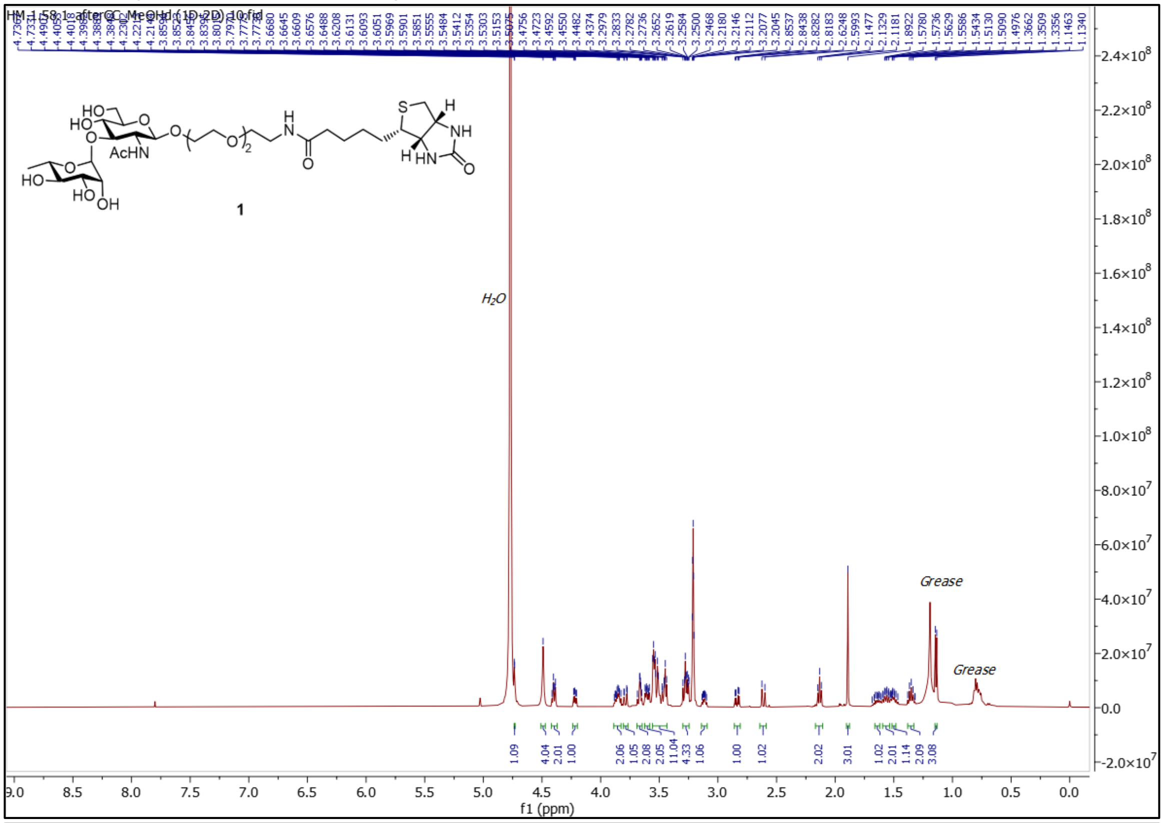

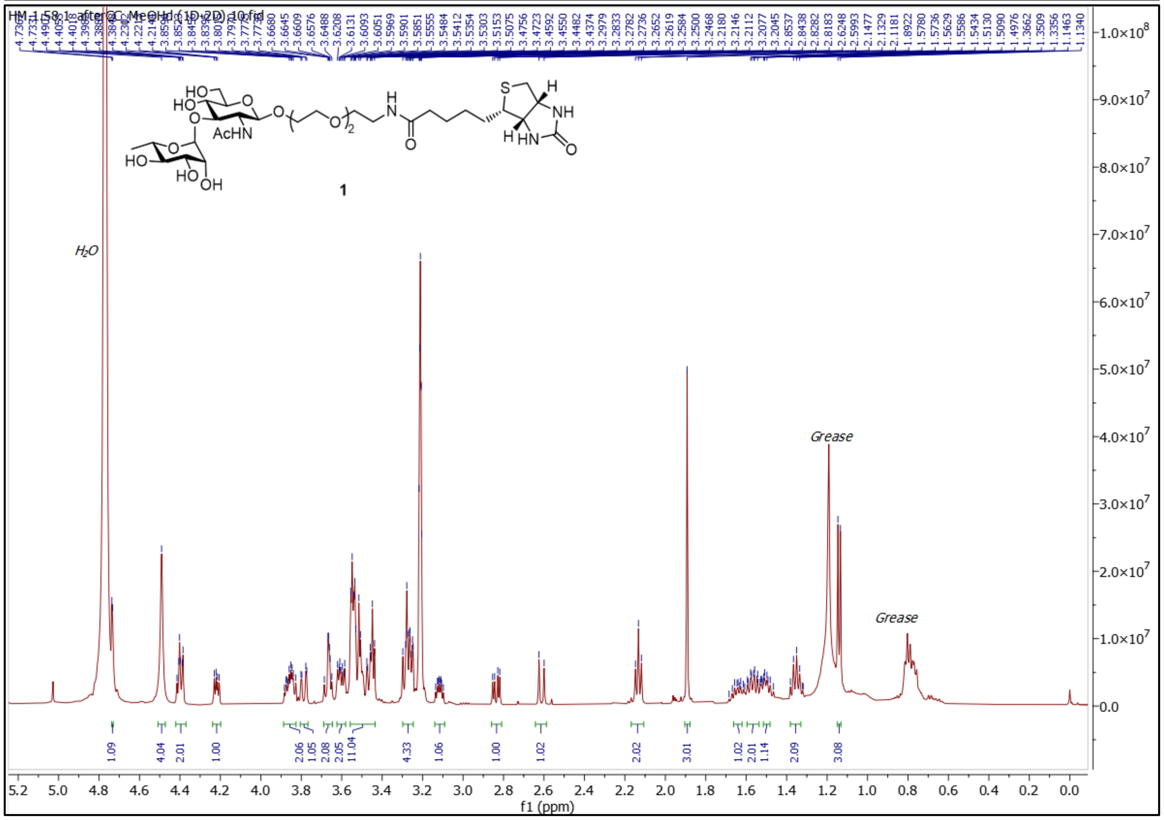

$^{13}C\{1H\}$ NMR (125 MHz, $CD_3OD$) Compound **1**:

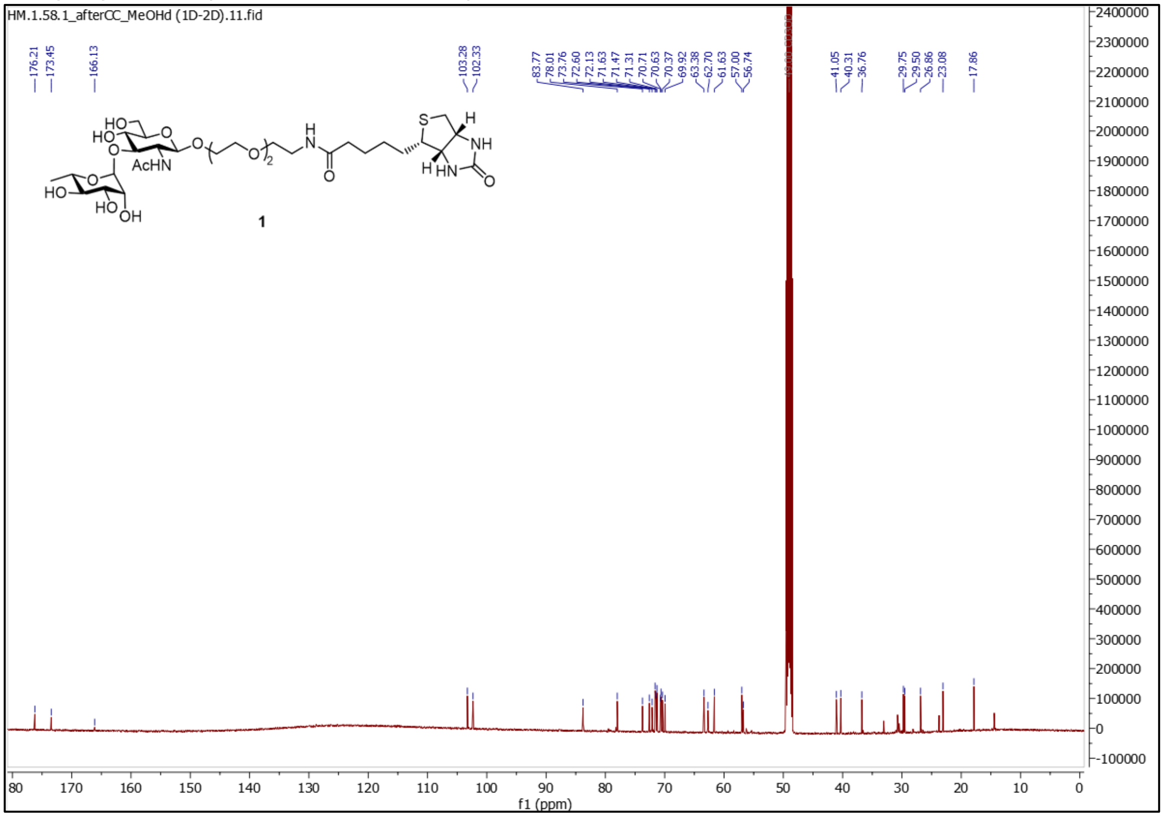

DEPT 135° compound **1**:

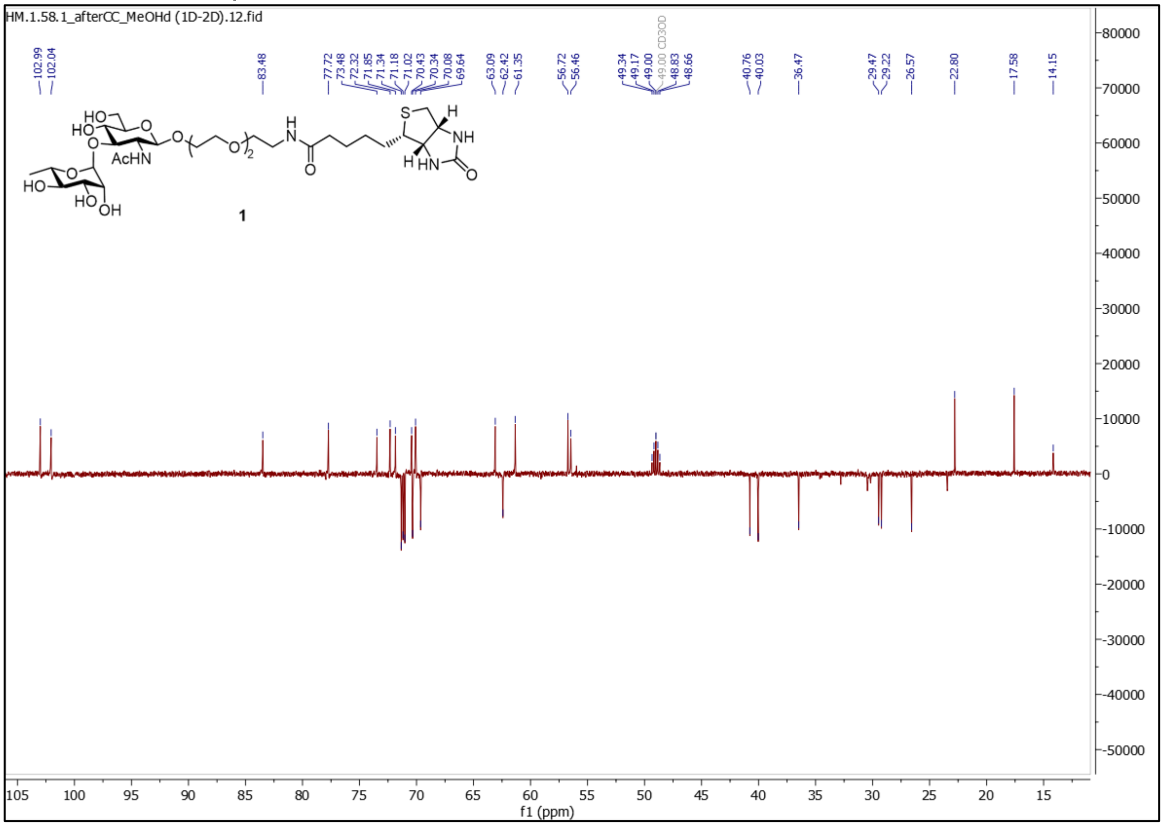

$^{1}H$-$^{13}C$ HSQC compound **1**:

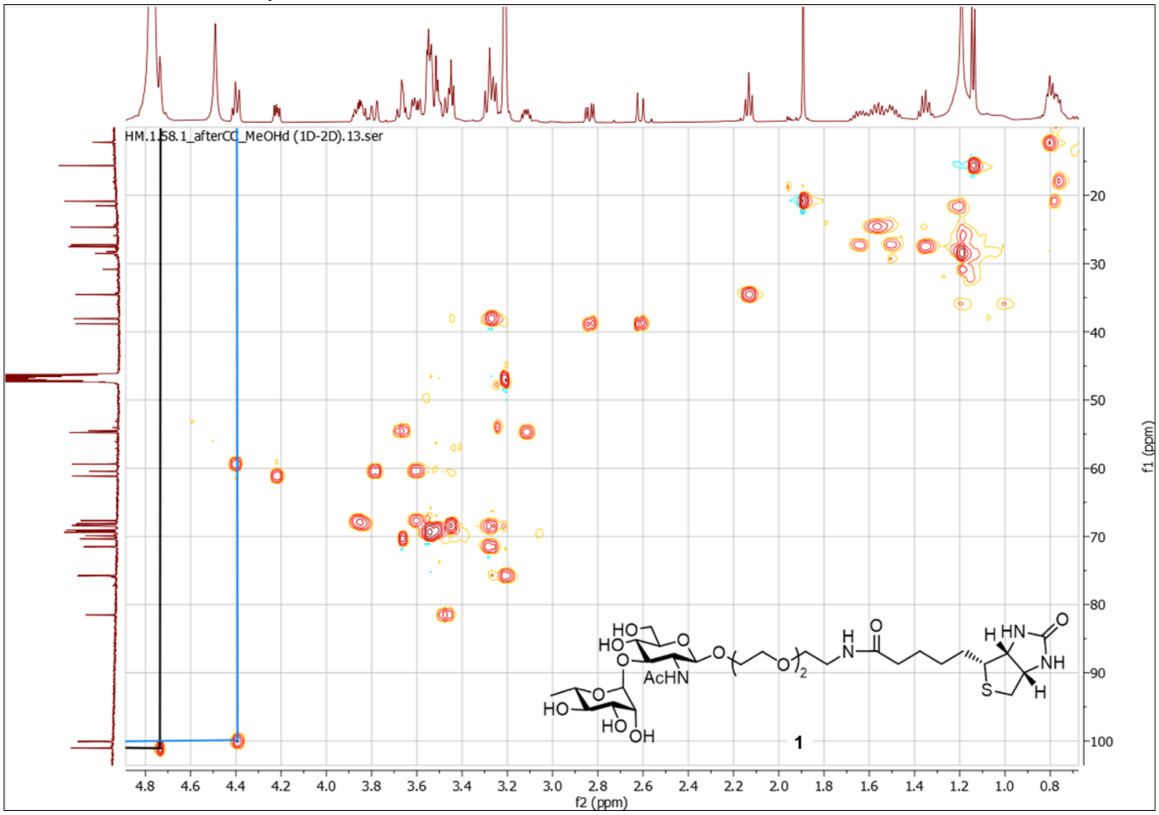


$^{1}H$-$^{13}C$ HMBC compound **1**:

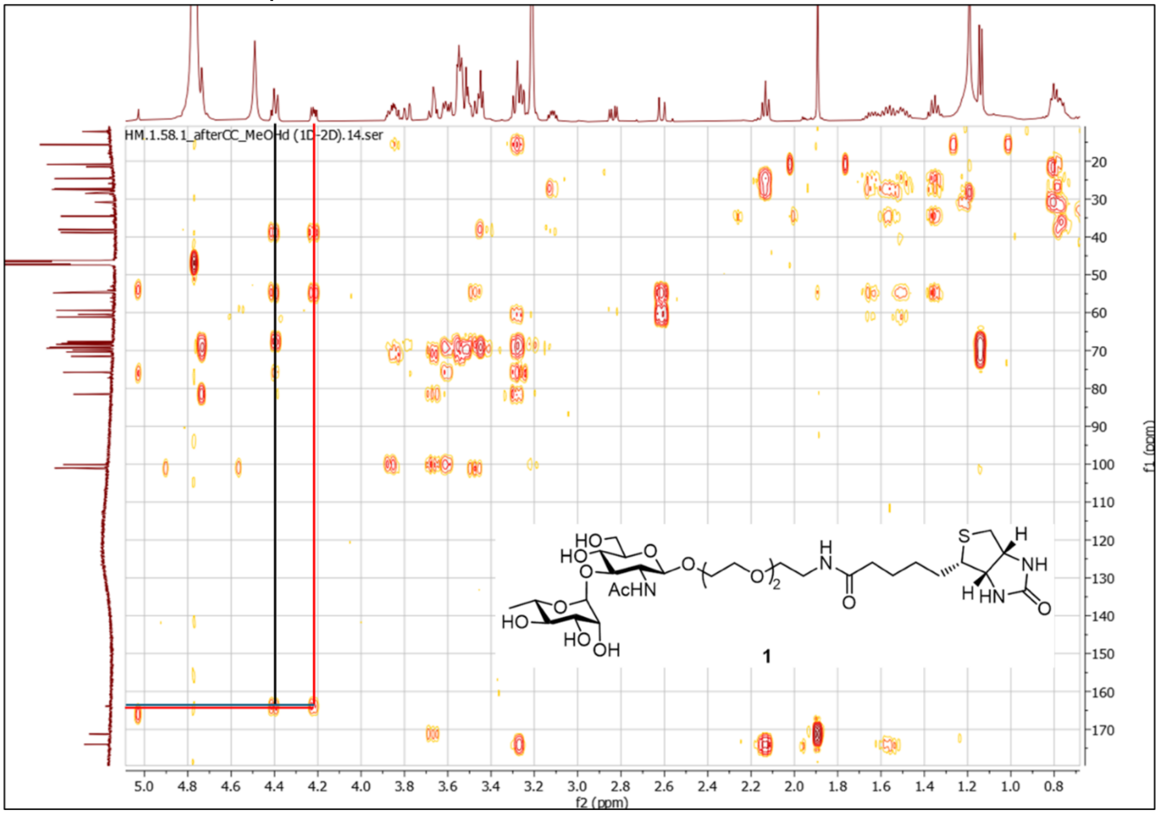

Supplement: Supplementary file 1 [file id5c00757_si_001.pdf]
